# Topological Dirac Semimetallic Phase in Heusler-Type Li_2_YZ (Y = Zn or Cd and Z = Ge, Sn, or Pb) Compounds: A First-Principles Investigation

**DOI:** 10.1021/acsomega.5c01191

**Published:** 2025-11-19

**Authors:** Fareeha Waheed, Ina Marie R. Verzola, Sreeparvathy Puthiya Covilakam, Rovi Angelo B. Villaos, Ancieto B. Maghirang, Zhi-Quan Huang, Feng-Chuan Chuang

**Affiliations:** † Department of Physics, 34874 National Sun Yat-Sen University, Kaohsiung 80424, Taiwan; ‡ Physics Division, National Center for Theoretical Sciences, Taipei 10617, Taiwan; § Center for Theoretical and Computational Physics, National Sun Yat-Sen University, Kaohsiung 80424, Taiwan; ∥ Department of Physics, National Tsing Hua University, Hsinchu 30013, Taiwan

## Abstract

Topological Dirac semimetals with robust surface states have drawn significant interest in condensed matter research, as they exhibit unique transport properties. Heusler compounds have emerged as an attractive platform offering a wide range of tunability in their electronic properties, ranging from semiconductors to semimetals, along with spin–orbit coupling-induced nontrivial topological states as well as crystalline symmetry-protected band crossings. These features position them as ideal candidates for investigating topological phases, including Dirac semimetallic behavior. In this study, we performed a first-principles investigation on Heusler-type Li_2_YZ (Y = Zn or Cd and Z = Ge, Sn, or Pb) compounds under two cubic space groups, F4̅3m (No. 216) and Fm3̅m (No. 225), to explore their electronic and topological properties, encompassing a total of 12 structures. Ground-state energy calculations identified the energetically preferred phase for each material. For brevity, Li_2_CdGe was selected as the representative compound, and its phonon dispersion spectra confirmed dynamic stability in both F4̅3m and Fm3̅m phases. Furthermore, the topological phase of Li_2_CdGe transitions from a triple-point phase to a fourfold Dirac node phase in both structures, F4̅3m and Fm3̅m, under spin–orbit coupling (SOC). The Dirac nature of Li_2_CdGe was validated by calculating its surface states on the (001) surface in both the F4̅3m and Fm3̅m phases. Notably, four of the studied compounds (Li_2_CdGe, Li_2_CdPb, Li_2_CdSn, and Li_2_ZnPb) exhibit nontrivial topological phases in two space groups. Our findings indicate that the Li_2_YZ family hosts promising nontrivial topological features to encourage further theoretical and experimental research in materials science and condensed matter.

## Graphical abstract

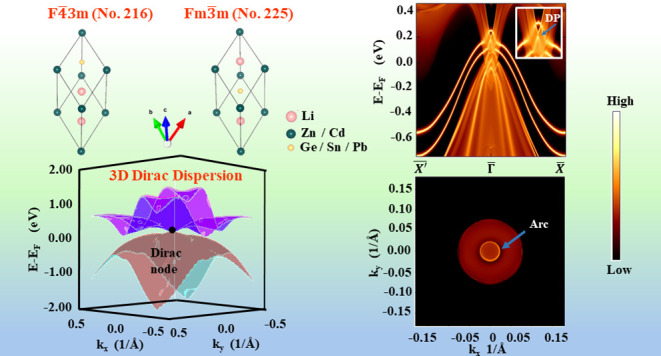

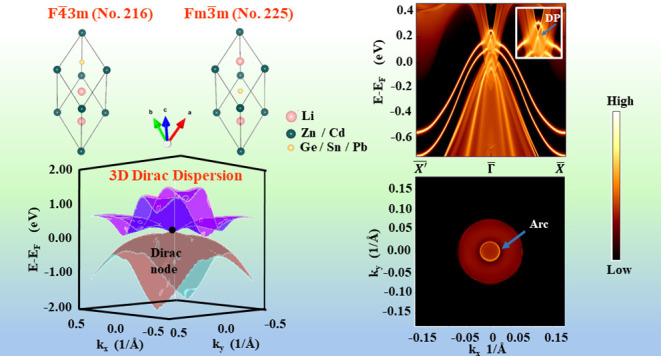

## 1 Introduction

Topological quantum materials (TMs) have transformed the dynamics of modern condensed matter physics and have prompted researchers to unveil their nontrivial characteristics^1,2^ owing to their exotic charge mobility, quantum states, and other optical properties.^3,4^ These topological materials are characterized by symmetry-protected surface states and nontrivial band topology.^1,3^ Formerly, the realization of topological insulators^5,6^ has led to the discovery of various classes of topological semimetals, such as Dirac semimetals,^7–9^ Weyl semimetals,^10–13^ nodal-line semimetals,^14–17^ and other materials of topological nature, such as topological crystalline insulators (TCIs).^18,19^ The advancement in recognizing new quantum phases in condensed matter has improved our understanding of symmetry-protected topological surface states in Dirac semimetals. Topological Dirac semimetals have been a subject of emphasis as they find potential applications in spintronic and low-power electronic devices^20,21^ and showcase remarkable physical phenomena such as quantum magnetoresistance,^22,23^ giant diamagnetism,^24,25^ and the oscillating quantum spin Hall effect.^26–28^

In particular, Dirac semimetals are characterized by fourfold band crossings near the Fermi level and host massless fermions in low-energy excitations, which give rise to remarkable physical properties such as high carrier mobility,^29^ the existence of Fermi arcs,^30^ and chiral anomaly.^31^ The quasiparticle excitation in Dirac semimetals due to two doubly degenerate bands crossing in crystalline materials with time-reversal symmetry (TRS) and inversion symmetry (IS) can be expressed by the relativistic Dirac equation, giving the name Dirac semimetal. The breaking of time-reversal symmetry (TRS) or inversion symmetry (IS) leads to the splitting of fourfold Dirac nodes into a pair of twofold Weyl nodes, which exhibit opposite chiralities.^32^ The first experimentally studied Dirac semimetal phase was reported in graphene, where the existence of two-dimensional (2D) Dirac fermions at the Fermi level was associated with unique transport properties, such as high electron mobility and the chiral quantum Hall effect.^33^ Comprehensive research on the 2D Dirac phase in graphene shifted the focus to realizing the Dirac semimetal state in three-dimensional (3D) systems.^34^ Following the investigation of Dirac semimetallic behavior in Cd_3_As_2_ ^23,35^ and 3D Dirac fermions in experimentally realized bulk Na_3_Bi,^9^ a large number of Dirac semimetals have been explored. The search for Dirac semimetals through angle-resolved photoemission spectroscopy (ARPES) investigations confirmed several transition metal dichalcogenides (TMDs) such as PtSe_2_,^36,37^ PtTe_2_,^38,39^ PdTe_2_,^40,41^ and NiTe_2_ ^42–45^ as type-II Dirac semimetals. Further, the existence of the Dirac phase has been reported in Ca-based Zintl compounds^46^ and in bulk Y_3_InC^47^ with the help of symmetry-protected surface states.

Meanwhile, a class of materials known as Heusler compounds has displayed a rich blend of electronic, magnetic, and topological properties.^48–57^ A theoretical investigation on Heusler compounds has shown the potential of half-Heusler alloys KXSb (X = Be, Mg, Ca, or Sr) to exhibit thermoelectric and optical properties.^58^ Furthermore, a theoretical study on the YPd_2_Sn-class^59^ belonging to the full-Heusler family has predicted the presence of type-II Dirac fermions in these materials. Another full-Heusler family XInPd_2_ (X = Ti, Zr, or Hf) has been reported to be type-II Dirac semimetals.^32^ Likewise, Heusler-type compound Ag_2_TmMg^60^ has been demonstrated to exhibit a Dirac state.

Studies on Heusler compounds have shown that they can undergo transitions from trivial to topological phases due to tunable lattice parameters, spin–orbit coupling (SOC), and strain engineering,^61^ offering opportunities for realizing quantum phenomena such as the quantized anomalous Hall effect^62,63^ and topological superconductivity.^64^ In particular, recent studies on half-Heusler TbPtBi have shown that SOC induces a phase transition from a metallic to a topological semimetallic phase, while compressive strain can drive further transitions from a topological semimetal to a trivial semimetal and eventually to a semiconductor.^51^ Furthermore, a family of half-Heusler compounds XYZ (X = Na or K; Y = Cu, Ag, or Au; Z = S, Se, or Te) has been investigated theoretically to reveal a phase transition from a trivial insulator to a topological insulator or Dirac semimetal based on the interaction between p and d states that eventually determines the SOC effect.^65^ Notably, Co_2_-based Heusler compounds, such as Co_2_TiSn^63^ and Co_2_MnGa,^66^ have been reported to present topological Dirac phases, where the inclusion of SOC plays a pivotal role in the Dirac point. These results highlight the critical role of SOC and strain in tuning topological phases in Heusler compounds, which is relevant to understanding the electronic properties of the Li_2_YZ (Y = Zn, Cd; Z = Ge, Sn, or Pb) series studied here.

Although numerous studies have reported the existence of the Dirac phase in Heusler compounds, the search for experimentally stable materials with promising Dirac features is still ongoing. Therefore, to explore Dirac band topology, we picked a Li-based Heusler family, with a general chemical formula Li_2_YZ (where Y = Cd or Zn and Z = Ge, Sn, or Pb), in which the compounds like Li_2_CdPb,^67^ Li_2_CdGe,^68^ Li_2_ZnGe,^68^ Li_2_ZnSn,^69^ and Li_2_CdSn^70^ have already been experimentally synthesized. Different studies exist on a few materials in this family, such as structural analysis of Li_2_CdPb,^67^ elastic and electronic properties of Li_2_ZnGe,^71^ and thermoelectric properties of Li_2_ZnGe,^72^ but the topological analysis of compounds with distinct electronic band structures and surface states is still lacking. Li_2_YZ materials are structurally identical to a series of Li-based compounds with the formula Li_2_M′X (where M′ = Cu, Ag, Au, or Cd and X = Sb, Bi, or Sn),^73^ which have been reported to manifest topological behavior by referring to a topological phase diagram. This indicates that the Li_2_YZ family can be a candidate for studying topological features.

Motivated by these previous findings, we investigated the structural, electronic, and topological properties of six Li_2_YZ (where Y = Cd or Zn and Z = Ge, Sn, or Pb) full-Heusler compounds in two different space groups, namely F4̅3m (No. 216) and Fm3̅m (No. 225), totaling 12 structures. These two different space groups are based on the experimentally synthesized structures^67–70^ of materials in this family. The electronic and topological properties of the six materials in two different structures F4̅3m (No. 216) and Fm3̅m (No. 225) are determined by the band structures obtained under PBE-GGA and hybrid functional HSE06. We present a comparative analysis of structural, electronic, and topological properties of Li_2_YZ compounds in two space groups F4̅3m (No. 216) and Fm3̅m (No. 225). For a detailed investigation of nontrivial topology, Li_2_CdGe has been regarded as a representative case, as it hosts a Dirac point close to the Fermi level. We verified the Dirac semimetallic phase in Li_2_CdGe in both space groups by the presence of surface states. Our results present an extended study on full-Heusler materials, where experimentally synthesized Li-based compounds show promising Dirac features, making them ideal for applications in electronic and topological devices.

## 2 Computational Details

The Vienna Ab initio Simulation Package (VASP)^74,75^ was used to perform first-principles calculations within the density functional theory framework^76^ with the projector-augmented-wave (PAW) method.^77,78^ The plane wave cutoff energy was set to 500 eV. All crystal structure relaxations were conducted until the residual force acting on each atom was less than 0.001 eV/Å using the Perdew–Burke–Ernzerhof (PBE) exchange–correlation functional within the generalized gradient approximation (GGA)^79^ exchange–correlation functional. Additionally, the requirements for self-consistent calculation convergence were set at 10^–6^ eV. The Γ-centered Monkhorst–Pack^80^ grid of 18 × 18 × 18 was used to model the first Brillouin zone. Also, the calculations performed in this study used the primitive unit cell to reduce the number of atoms. The PBE-GGA functional was used to calculate the total ground state energy and band structure. Additionally, the Heyd–Scuseria–Ernzerhof hybrid functional (HSE06)^81–83^ through a reduced Γ-centered Monkhorst–Pack grid of 9 × 9 × 9 was further implemented to get more accurate electronic properties.^80,84–86^ The topological properties were evaluated using the SymTopo^87^ package, which uses symmetry-based indicators that can categorize different topological properties, such as trivial insulator (I), conventional metal (M), topological insulator (TI), topological crystalline insulator (TCI), high-symmetry-point semimetal (HSPSM), and high-symmetry-line semimetal (HSLSM). Moreover, the maximally localized Wannier functions (MLWFs) were obtained from the Wannier90 ^88^ program, and the surface states were determined using surface Green’s function as implemented in the WannierTools^89^ program. Phonon dispersion spectra were obtained through the Phonopy^90^ package using a 3 × 3 × 3 supercell. Lastly, further confirmation of topological properties was obtained by calculating the *Z*_2_ topological invariants through Fu-Kane parity analysis^91^ and the evolution of Wannier charge centers (WCCs) obtained by using WannierTools.^89^ For 3D materials, the strong and weak topological indices are represented as (ν_0_;ν_1_ν_2_ν_3_), where ν_0_ denotes the strong topological index and ν_1_, ν_2_, and ν_3_ are the weak indices.^91^ With the calculations under WannierTools, a strong topological index is determined using the relation ν_0_ = [*Z*_2_(*k*_*i*_ = 0) + *Z*_2_(*k*_*i*_ = 0.5)]­mod2, while the weak indices are given by ν_*i*_ = *Z*_2_(*k*_*i*_ = 0.5), where *i* = 1, 2, 3.

## 3 Results and Discussion

### 3.1 Structural Geometry and Stability

The Li_2_YZ (Y = Cd, Zn and Z = Ge, Sn, or Pb) compounds crystallize in a cubic structure belonging to the full-Heusler family. The members of this family have been reported to crystallize in two space groups, F4̅3m (No. 216) and Fm3̅m (No. 225),^67–70^ both of which are different phases of the cubic structure. Specifically, the crystal structures of F4̅3m (No. 216), in conventional and primitive unit cells, are depicted in Figure 1a–d. The crystal structure belongs to the *T*_d_ point group, which exhibits three rotational symmetries of *C*_2_, *C*_3_, and *C*_4_. On the other hand, the Fm3̅m structure in its conventional and primitive unit cells is represented in Figure 1e–h. The unit cell of the Li_2_YZ Fm3̅m (No. 225) phase demonstrates a NaCl-type crystal structure where two Li atoms are located as nearest neighbor atoms. This crystal structure belongs to the point group O_h_ and exhibits mirror symmetries *IC*_4_ and *IC*_2_ and *C*_2_, *C*_3_, and *C*_4_ rotational symmetries. Moreover, Fm3̅m possesses greater symmetry than space group F4̅3m due to the additional mirror symmetries via slight distortion of atomic positions.^92^ In cubic Heusler structures, Fm3̅m corresponds to the regular Heusler structure with higher symmetry, while F4̅3m represents the inverse Heusler structure with reduced symmetry.

**Figure 1:**
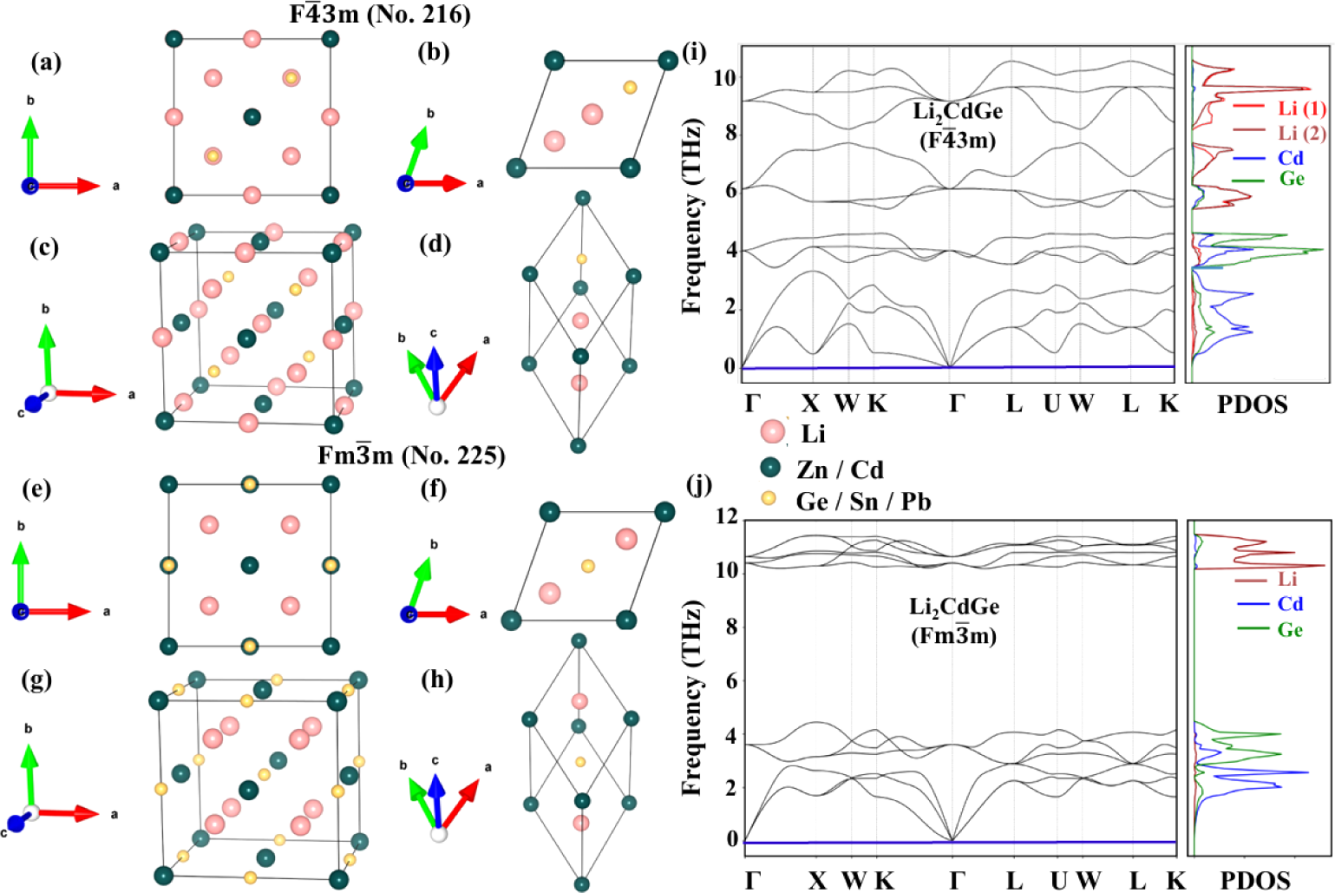
Top view of F4̅3m (No. 216) in the (a) conventional unit cell and (b) primitive unit cell. The perspective view of F4̅3m (No. 216) in the (c) conventional unit cell and (d) primitive unit cell. The top view of Fm3̅m (No. 225) in the (e) conventional unit cell and (f) primitive unit cell. The perspective view of Fm3̅m (No. 225) in the (g) conventional unit cell and (h) primitive unit cell. The pink spheres represent Li, the teal spheres represent Cd or Zn, and the golden spheres represent Z = Ge, Sn, or Pb elements. Phonon dispersion spectra with partial phonon density of states (PDOS) of Li_2_CdGe in (i) F4̅3m and (j) Fm3̅m phases.

The difference between two cubic phases F4̅3m (No. 216) and Fm3̅m (No. 225) can be easily seen by comparing the top view representation of the conventional and primitive unit cell in Figure 1a,b for space group F4̅3m (No. 216) with Figure 1e,f for space group Fm3̅m (No. 225). Here, space group Fm3̅m (No. 225) has more symmetrically oriented placements of Li, Cd/Zn, and Ge/Sn/Pb atoms. Additionally, in the Fm3̅m structure, Li, Cd, and Ge atoms are symmetrically arranged, with each Li atom positioned between two Cd atoms and Ge occupying the center of the primitive unit cell, as shown in Figure 1h. Moreover, in the Fm3̅m phase, the bonds are more symmetric, with uniform Li–Cd (2.79 Å) and Li–Ge (2.79 Å) lengths, while Cd–Ge bonds (3.22 Å, 5.58 Å) exhibit greater symmetry, as given in Table S1. Contrastingly, in the F4̅3m structure, Ge is displaced from the center, and Li is positioned unevenly relative to Cd in the primitive unit cell, as given in Figure 1d. This asymmetry leads to nonuniform bond lengths in the F4̅3m phase, with variations in Li–Cd (3.25 Å, 2.81 Å), Li–Ge (3.25 Å, 2.81 Å), and Cd–Ge (2.81 Å, 5.39 Å), as summarized in Table S1.

Furthermore, the total ground state energy calculations for all six materials in both F4̅3m and Fm3̅m space groups are consolidated in Table S2. Four compounds (Li_2_CdGe, Li_2_ZnPb, Li_2_ZnGe, and Li_2_ZnSn) have lower ground-state energies in the F4̅3m structure, while Li_2_CdPb and Li_2_CdSn exhibit lower energies in the Fm3̅m structure. However, the energy differences between these two structures for all six compounds range from approximately 0.001 to 0.364 eV, rendering their ground-state energies comparable. Notably, in Table S2, it can be seen that Li_2_CdPb and Li_2_CdSn adopt Fm3̅m structure, while Zn-based compounds (Li_2_ZnPb, Li_2_ZnGe, and Li_2_ZnSn) prefer the F4̅3m phase. This structural preference is a consequence of atomic size mismatch, bonding preferences, and lattice strain. In Li_2_CdPb and Li_2_CdSn, large atomic sizes of Cd (1.48 Å), Sn (1.45 Å), and Pb (1.54 Å) reduce size mismatch, thus minimizing the lattice strain, where Cd–Sn and Cd–Pb interactions (Cd(s)–Pb/Sn­(p) orbital hybridization) stabilize the symmetric cubic phase Fm3̅m.

While in Li_2_CdGe, smaller atomic radius (1.25 Å) of Ge and the large atomic size of Cd (1.48 Å) lead to local strain in the symmetric Fm3̅m phase, in the Zn-based compounds (Li_2_ZnPb, Li_2_ZnGe, and Li_2_ZnSn), contracted 4s orbitals and a smaller atomic radius (∼1.39 Å) of Zn result in weaker orbital overlap with the p orbitals of the Z (Ge, Sn, and Pb) atoms. This introduces internal strain in the high-symmetry Fm3̅m structure.

In response to the internal strain, Li_2_CdGe and Zn-based systems (Li_2_ZnPb, Li_2_ZnGe, and Li_2_ZnSn) reduce their total energy by distorting to F4̅3m, where Li atoms occupy two inequivalent sites, forming Li–Zn_4_/Cd_4_ and Li–Ge_4_/Sn_4_/Pb_4_ tetrahedra. This leads to a more flexible lattice where slight distortions in atomic positions reduce lattice strain and lower the system’s total energy, making it more energetically favorable in F4̅3m.

Such symmetry-lowering transitions have previously been studied in Heusler systems, where atomic size mismatch induces internal strain and the structure transforms into a lower symmetry modulated structure.^93–95^

Due to the structural similarity and comparable ground state energies of two space groups F4̅3m and Fm3̅m, we examined the dynamic stability and electronic properties of Li_2_CdGe in both structural phases. Figure 1i,j indicates the phonon dispersion curves for Li_2_CdGe in F4̅3m and Fm3̅m phases, wherein no imaginary frequencies in phonon spectra imply that Li_2_CdGe is dynamically stable in both space groups. Comparing the phonon spectra of Li_2_CdGe in F4̅3m and Fm3̅m in Figure 1i,j reveals notable differences arising from their structural symmetries. In the higher symmetry Fm3̅m phase, Figure 1j displays smooth dispersion of acoustic phonon modes around the Γ point, and optical phonon modes exhibit degeneracy due to the overlap of vibrational frequencies. This degeneracy results from multiple vibrational modes transforming similarly under the symmetry operations of the Fm3̅m group, leading to overlapping phonon bands. In contrast, the lower symmetry of the F4̅3m phase causes the splitting of phonon modes, resulting in sharper bands with observable gaps between phonon branches, as shown in Figure 1i. The relaxation of the symmetry constraints in this phase gives rise to distinct dispersion curves. These differences can be attributed to the variations in atomic arrangements, as shown in Figure 1a–h, and bond lengths between the two space groups, as represented in Table S1. To examine the role of atomic vibrations in phonon spectra, we also examined the partial phonon densities of states (PDOS) along with full phonon dispersion, as shown in the left panel of Figure 1i,j. The distribution of phonon modes reveals that Li atoms with a lower atomic mass compared to Cd and Ge predominantly dominate in the higher frequency region; on the other hand, Cd and Ge contribute to the lower frequency range. In the PDOS spectra of Li_2_CdGe in the F4̅3m phase, the distributions of Li atoms have a double representation (represented by red and magenta bands in the left panel of Figure 1i. This is attributed to the reduced crystal symmetry and local atomic bonding in space group F4̅3m, where Li atoms occupy two inequivalent sites, forming Li–Cd_4_ and Li–Ge_4_ tetrahedra, leading to different PDOS profiles.

Additionally, Figure S1 illustrates the phonon dispersion spectra with a partial phonon density of states for the five compounds (Li_2_CdPb, Li_2_CdSn, Li_2_ZnGe, Li_2_ZnPb, and Li_2_ZnSn) in their energetically preferred phases. The absence of imaginary frequencies in the phonon dispersion curves of these five compounds indicates that they are dynamically stable.

Furthermore, Table 1 summarizes the optimized lattice constants of the Li_2_YZ compounds in their energetically preferred phases. The calculated lattice constants show good agreement with the experimental values.

**Table 1:** Optimized Lattice Constants of Li_2_YZ Compounds in Their Low-Energy Structural Phase under PBE-GGA and the Comparison with Experimental Lattice Constants

		Optimized Lattice Constant (*a* = *b* = *c*) Å		
Materials	Energetically Preferred Phase	Primitive Cell	Conventional Cell	Experimental Lattice Constant (*a* = *b* = *c*) Å
**Li**_**2**_ **CdGe**	F4̅3m **(216)**	4.599	6.508	6.410 ^68^
**Li**_**2**_ **CdPb**	Fm3̅m **(225)**	4.820	6.862	6.837 ^67^
**Li**_**2**_ **CdSn**	Fm3̅m **(225)**	4.747	6.733	6.727 ^70^
**Li**_**2**_ **ZnGe**	F4̅3m **(216)**	4.359	6.158	6.140 ^68^
**Li**_**2**_ **ZnPb**	F4̅3m **(216)**	4.732	6.616	N/A
**Li**_**2**_ **ZnSn**	F4̅3m **(216)**	4.607	6.485	6.650 ^69^

### 3.2 Electronic Properties of Li_2_CdGe in Space Groups F4̅3m and Fm3̅m

After determining the energetically preferred structures of the Li_2_YZ compounds, we explored their electronic properties. Since the ground state energy comparison of the two space groups F4̅3m and Fm3̅m indicates that both structures have comparable ground state energies and have a minimal difference in atomic arrangements, then the two structural phases F4̅3m and Fm3̅m can be interconvertible under rotational distortions along a certain axis.^92^ To explore the possible phase transformation, we subjected Fm3̅m structure (Li_2_CdGe) to a 20° atomic rotation about the [001] axis using VASP. After the structural optimization, symmetry analysis with Phonopy verified that the distorted structure stabilized in the F4̅3m phase. Moreover, 3% strain along the *c*-axis to Fm3̅m structure generated another distorted structure, I4̅3m, which further validates the transformation mechanism and supports our interpretation that such transitions are possible under symmetry-oriented distortions. This observation indicates that the two cubic phases can be interconverted through symmetry-breaking rotational distortion^88^ and highlights the structural flexibility of these compounds under suitable conditions.

Thus, by considering the possibility of the transformation of one phase into the other under symmetry-oriented rotations, we have plotted the band structures of Li_2_CdGe in both space groups (F4̅3m and Fm3̅m). Figure 2a and b represent the band structure of Li_2_CdGe under PBE-GGA without and with SOC, respectively, in the F4̅3m space group. The PBE-GGA band structures of Li_2_CdGe in the Fm3̅m space group are given in Figure 2e and f without and with SOC, wherein the Dirac point is located at *E* – *E*_F_ = 0.350 eV. The orbital band projections of Li_2_CdGe in two space groups, F4̅3m and Fm3̅m, are represented in Figure 2c,d and g,h. The band structures of Li_2_CdGe show similar properties at the Γ point in both structural phases (F4̅3m and Fm3̅m), wherein the band structures show a triply degenerate crossing point at Γ without SOC, while fourfold degenerate band crossings can be seen at Γ under the SOC effect. By comparing the band structures in the two phases (F4̅3m and Fm3̅m), we observed slight differences in the band profile along the high-symmetry path shown in Figure 2. Focusing on the band structure for space group F4̅3m, as shown in Figure 2b, split and sharper bands are observed above the Fermi level at high-symmetry points X and Γ. The location of the Dirac point is at *E* – *E*_F_ = 0.189 eV. The orbital band projections of Li_2_CdGe (F4̅3m) in Figure 2d show that the band splitting at X is contributed by s orbitals from Cd, while p orbitals from Cd and Ge govern the splitting at Γ. Considering the band structure for space group Fm3̅m, depicted in Figure 2f, degenerate and broader bands have been observed at high-symmetry points X, Γ, and L. These bands arise due to the overlapping of degenerate states contributed by p orbitals from Cd and Ge, as evidenced by Figure 2h.

**Figure 2:**
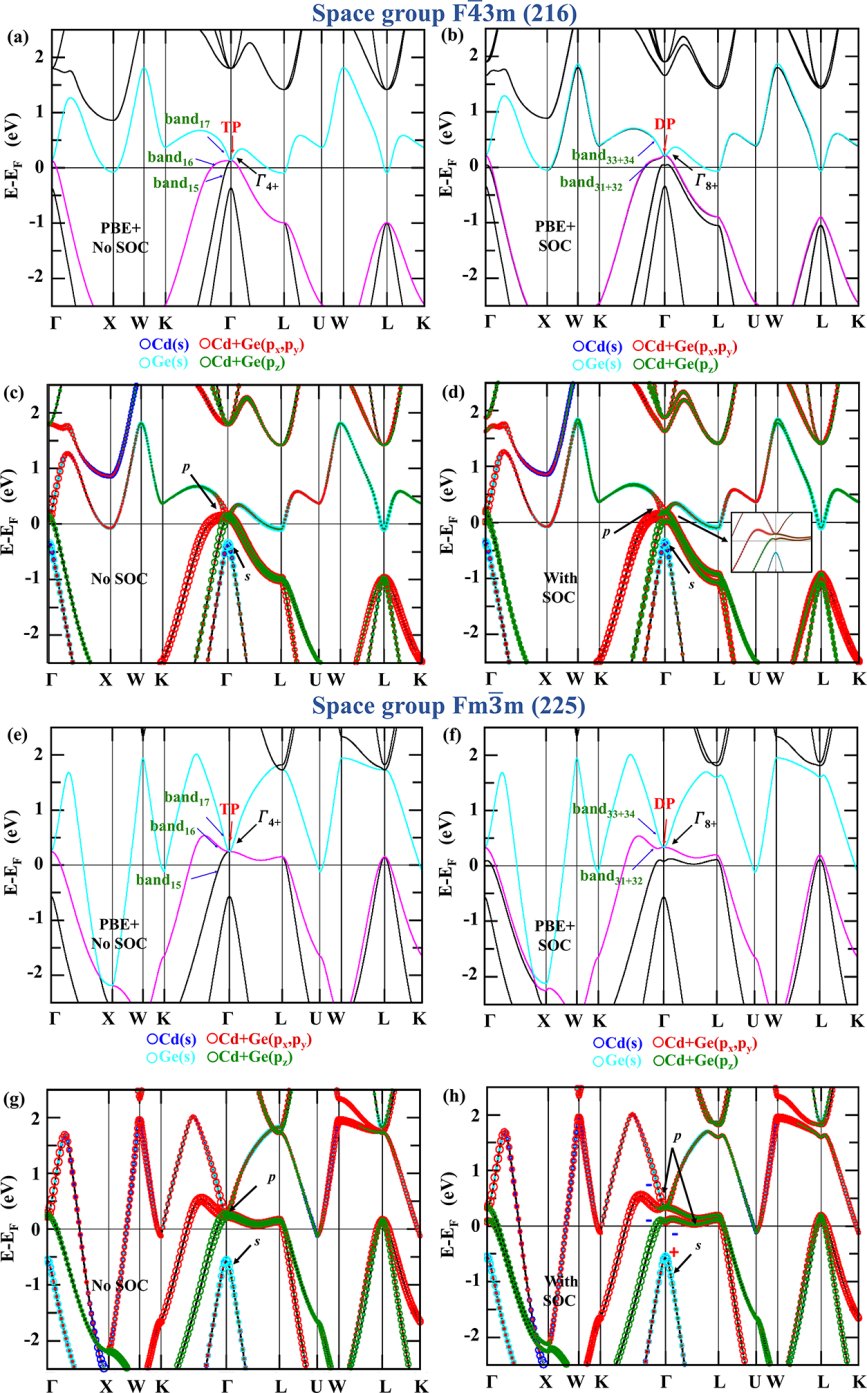
Band structure of Li_2_CdGe in the F4̅3m (216) phase for (a) without SOC and (b) with SOC under PBE-GGA. Red arrows indicate (a) triple-point (TP) and (b) Dirac point (DP). Orbital band projections of Li_2_CdGe (c) without SOC and (d) with SOC in the F4̅3m (216) phase (inset shows the zoomed-in view of degenerate bands at the Γ point). Blue and cyan circles represent s orbitals from Cd and Ge, respectively; red circles represent p_*x*_ and p_*y*_ states, and green circles represent *p*_*z*_ states from Cd and Ge, respectively. Black arrows point at s and p orbitals, where the position of s orbitals under p orbitals is an indication of the reordering of atomic orbitals with and without SOC. Band structure of Li_2_CdGe in the Fm3̅m (225) phase (e) without SOC and (f) with SOC under PBE-GGA. Red arrows indicate (e) triple-point (TP) and (f) Dirac point (DP). Orbital band projections of Li_2_CdGe (g) without SOC and (h) with SOC in the Fm3̅m (225) phase. Even (+) and odd (−) parities of eigenstates at Γ are indicated by signs. Blue and cyan circles represent s orbitals from Cd and Ge, respectively; red circles represent p_*x*_ and p_*y*_ states; and green circles represent *p*_*z*_ states from Cd and Ge, respectively.

The differences in the band structure between the two phases can be attributed to their unique symmetry-induced orbital interactions. The lower symmetry in the F4̅3m phase allows for more localized contributions from the s orbitals at X and L high-symmetry points. In contrast, the higher symmetry of the Fm3̅m phase promotes broader contributions from p orbitals.

The orbitally resolved band structures reveal the energetic ordering of atomic orbitals near the Fermi level, providing critical insight into the emergence of nontrivial topological characteristics. Figure 2c,d and g,h illustrate a clear manifestation of intrinsic inversion of atomic orbitals in Li_2_CdGe (F4̅3m and Fm3̅m phases) through the interplay between s and p states. As represented by arrows in Figure 2c,d, the s orbitals are positioned at lower energies (approximately −0.6 eV) relative to p (p_*x*_, p_*y*_, and p_*z*_) orbitals located at higher energies (around 0.3 eV), indicating that the system is nontrivial with an inherent band inversion even in the absence of SOC. This energy configuration contrasts with that of conventional trivial semiconductors, where the *s*-like conduction band states typically reside above the p-like valence states, representing an inversion order of band.^96^ Such inversion of orbital characters in topological materials is intimately tied to the nature of bonding of adjacent atoms in the crystal lattice.^96^ The presence of heavy elements like Cd and Ge increases the effective mass at the Fermi level, which subsequently shifts s orbitals to a reduced energy level while elevating the p orbitals, thereby facilitating the conditions necessary for band reordering.

As Li_2_CdGe energetically prefers the F4̅3m space group, a comprehensive discussion on hybrid functional, surface state calculations, and the evolution of Wannier charge centers (WCCs) has been executed in this structural phase. To explore the phase transition under the impact of spin–orbit coupling (SOC) on the Li_2_CdGe compound crystallizing in the F4̅3m space group, we analyze the band dispersion along the high-symmetry K−Γ–L path, as shown in Figure 3a,b. First, we discuss the mechanism behind the formation of a triple-point in the band structure of Li_2_CdGe without SOC. Figure 3a shows that a nondegenerate band meets a doubly degenerate band above the Fermi level at the Γ point. This leads to the generation of a triple-point, which is contributed by touching two degenerate bands 15 and 16 with a nondegenerate band 17. These threefold degenerate bands possess Γ_4+_ irreducible representation, which is three-dimensional, and the triple-point holds *C*_*2*_, *C*_*3*_, and *C*_*4*_ rotational symmetries. Furthermore, the analysis of orbital band projections in the absence of SOC depicts that p (p_*x*_, p_*y*_, and p_*z*_) orbitals of Cd and Ge dominantly contribute to the formation of TP near the Fermi level at Γ, as shown in Figure 3a.

**Figure 3:**
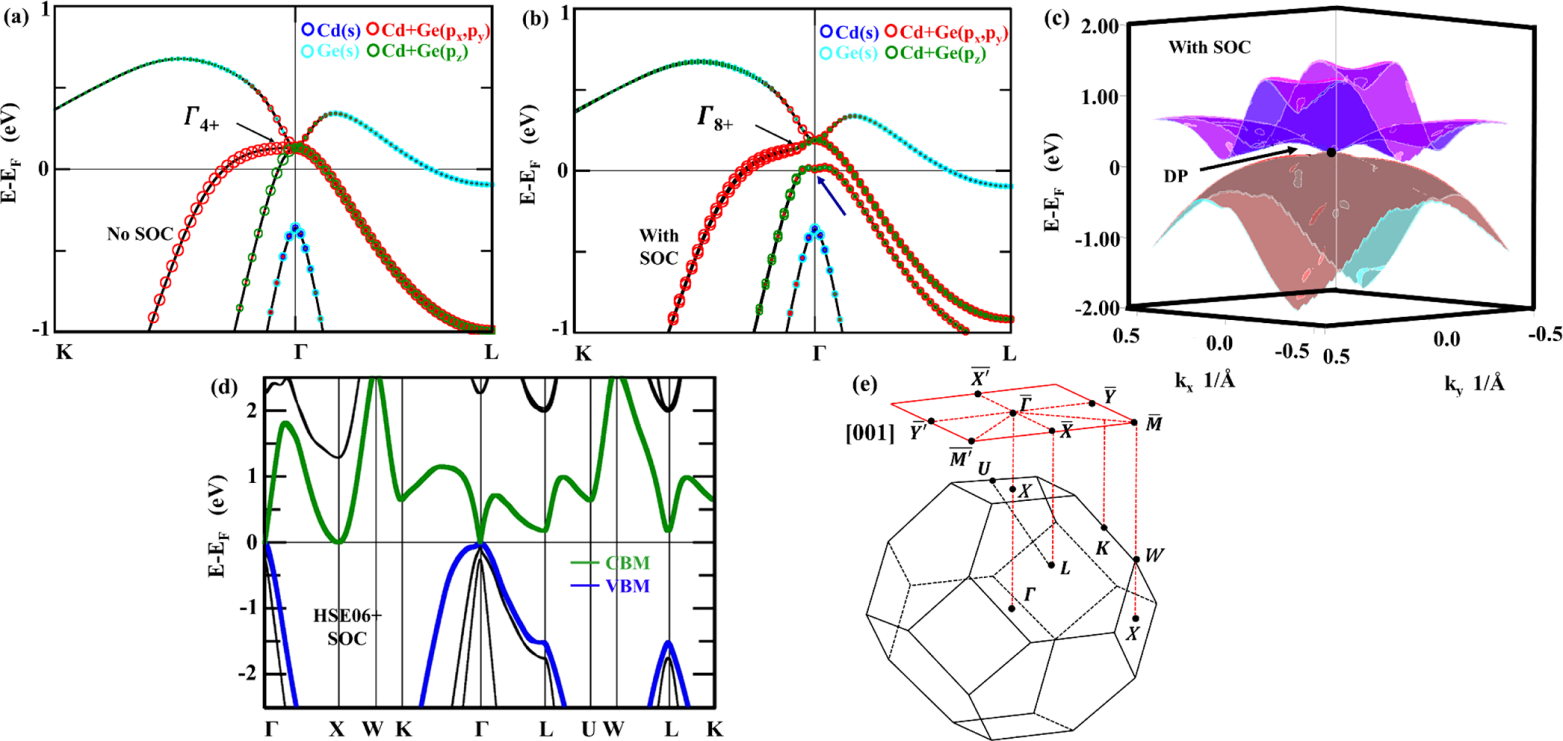
Orbital projected band structure of Li_2_CdGe in the F4̅3m (216) phase for (a) without SOC and (b) with SOC with irreducible representation, under PBE-GGA. Blue and cyan circles represent s orbitals from Cd and Ge, respectively; red circles represent p_*x*_ and p_*y*_ states, and green circles represent p_*z*_ states from Cd and Ge, respectively. The black arrows represent irreducible representations at Γ to generate TP/DP. The blue arrow shows band inversion after SOC. (c) The energy dispersion of a fourfold Dirac point obtained with SOC under PBE-GGA, with a black dot representing the Dirac point. Blue, magenta, red, and cyan-colored bands represent four bands. (d) HSE06 band structure of Li_2_CdGe in F4̅3m. Green and blue bands show the conduction band minimum and valence band maximum, respectively. (e) The first Brillouin zone of Li_2_YZ materials with the (001) surface-projected Brillouin zone.

A significant change in the band structure was observed upon inclusion of SOC. With SOC) the initially degenerate bands at the Γ point exhibit notable splitting and reordering. Figure 3b indicates the band structure of Li_2_CdGe with SOC along the high-symmetry points K−Γ–L. As indicated by the blue arrow in Figure 3b, p_*x*_, p_*y*_, and p_*z*_ orbitals of Cd and Pb contribute to the band inversion that generates a distinct curved-inverted warped structure centered at the Γ point. Furthermore, it has been observed that the band crossing at Γ under SOC involves the crossing between two doubly degenerate bands (31 + 32 and 33 + 34), forming a Dirac point. This SOC-driven inversion signals a topological phase transition, wherein the system evolves from a triple-point semimetal phase in the absence of SOC to a Dirac semimetal phase upon its inclusion. Similar behavior has been reported in Heusler compounds such as Co_2_TiSn^62^ and Co_2_MnGa,^66^ where SOC induces a topological phase transition from a triple-point to a Dirac point. The key orbitals playing a role in touching at the Γ high-symmetry point are the p (p_*x*_, p_*y*_, and p_*z*_) orbitals of Cd and Ge, as indicated in Figure 3b.

Symmetry analysis signifies that the Dirac point is protected by *C*_*2*_, *C*_*3*_, and *C*_*4*_ rotational symmetries, and the irreducible representation at this point is Γ_8+_. Thus, SOC lifts degeneracy and induces band inversion that leads to a topological phase transition from triple-point Fermion (TP) at Γ into a fourfold degenerate Dirac point (DP) in Li_2_CdGe.

A similar phenomenon of band inversion and SOC-induced topological phase transition (from triple-point Fermion to Dirac point) has been observed in Li_2_CdGe in space group Fm3̅m (225), as shown in Figures 2g,h and S17d–e, where the orbital-resolved band structures confirm consistent behavior. The band crossing at the Γ point involves a transition of irreducible representation, from Γ_4–_ to Γ_8+_ upon inclusion of SOC [given in Figure S17d-e]. Our symmetry analysis highlights that in addition to rotational symmetries (*C*_*2*_, *C*_*3*_, and *C*_*4*_), the Fm3̅m (225) space group possesses additional inversion symmetry (*I*), characterized by elements such as *IC*_*4*_ and *IC*_*2*_ *′*, which are absent in the F4̅3m (216) structure.

The existence of a Dirac node in Li_2_CdGe at Γ is further verified by 3D band structure calculation for F4̅3m (216) structure under PBE-GGA with SOC, as shown in Figure 3c. It is observed that the Dirac point is contributed by a fourfold degenerate crossing formed by two double-degenerate bands, with distinct color coding used to represent the band multiplicity. Figure 3d represents the band structure of Li_2_CdGe (F4̅3m space group) under the hybrid functional calculation (HSE06+SOC), which is consistent with the band structure under PBE-GGA shown in Figure 2b. The electronic band structures depict a band crossing between the conduction band minimum (CBM) and the valence band maximum (VBM), which lie close to the Fermi level. Figure 3e displays the bulk first Brillouin zone (BZ) of Li_2_YZ crystals in 3D with the surface-projected BZ along (001), laying the groundwork for further analysis of topological surface states.

The band structures of Li_2_CdGe (F4̅3m space group) under PBE-GGA and HSE06 give similar results regarding topological semimetallic features and band inversion near the Dirac point.^23,32^ This agreement confirms that PBE-GGA is sufficient to capture essential topological features, such as Dirac crossings and band inversion, in semimetallic systems. For Heusler-type compounds, multiple studies have demonstrated that PBE-GGA reliably identifies the nontrivial band topology and Dirac semimetallic behavior.^35,60^ Therefore, PBE-GGA remains a computationally efficient and accurate first-principles method for the investigation of topological semimetals, particularly in systems where the electronic structure is governed by symmetry-protected band crossings rather than subtle exchange–correlation effects, as previously been reported.^35,60,97^ For the family of Li_2_YZ compounds, both PBE-GGA and HSE06 provide reliable predictions of the band structure and electronic properties, and the choice of functional does not lead to a significant change in the ground state. Based on this agreement, we have performed surface state calculations using the PBE-GGA functional. Furthermore, an extra confirmation of nontrivial nature of Li_2_YZ materials was achieved by parity analysis based on Fu-Kane model^91^ and evolution of Wannier Charge Centers (WCCs) by using WannierTools.^89^ Since the cubic space group F4̅3m (No. 216) is non-centrosymmetric and lacks inversion symmetry,^98,99^ therefore parity eigenvalues were not defined in this case, thus parity analysis was only performed for Li_2_YZ materials in the centrosymmetric Fm3̅m (225) cubic phase. For the representative Li_2_CdGe in space group F4̅3m Z_2_ topological invariant was determined by using WCC evolution, where as for Li_2_CdPb in centrosymmetric Fm3̅m phase, both Fu-Kane parity analysis^100^ and WCC evolution^89^ were used.

### 3.3 Surface States of Li_2_CdGe in Space Group F4̅3m

Dirac semimetals carry the imprint of bulk degenerate bands on their surfaces. To capture the topological surface states, we have computed the surface-state spectra of Li_2_CdGe on the (001) surface under the PBE-GGA functional with SOC by Green’s function. Figure 4a–c shows the surface states for Li_2_CdGe in space group F4̅3m, calculated along three different high-symmetry paths: *X̅*’–*Γ̅*–*X̅*, *M̅*’–*Γ̅*–*M̅*, and *Y̅*’–*Γ̅*–*Y̅*. Figure 4a presents the surface states along the *X̅*’–*Γ̅*–*X̅*, path wherein two surface states marked by SS_1_ and SS_2_ arise from the Dirac point present at Γ and extend toward the *Γ*–*X̅* direction. Focusing on the surface-state spectra along the path *X̅*’–*Γ̅*–*X̅* in Figure 4a, it can be seen that topological surface states marked by SS_1_ arising from the valence band and SS_2_ originating from the conduction band make a Dirac cone at 0.189 eV. This energy range of the Dirac point at Γ is consistent with the electronic band structure given in Figure 2b, where a Dirac cone is formed by the crossing of valence and conduction bands at *E* – *E*_F_ = 0.189 eV above the Fermi level.

**Figure 4:**
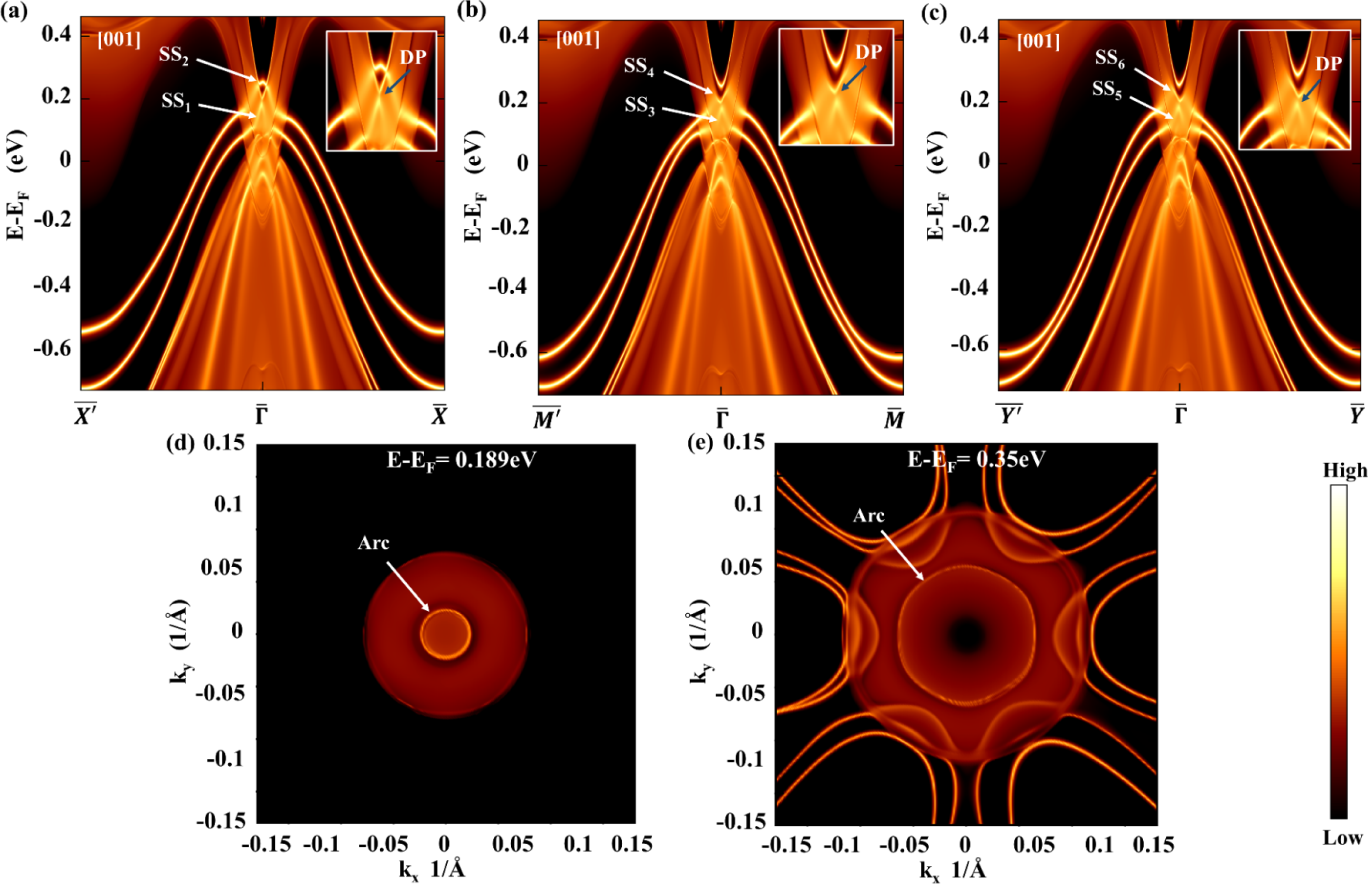
(a) Surface electronic spectrum for Li_2_CdGe in the F4̅3m (216) phase showing the topologically protected surface states along (a) *X̅*’–*Γ–X̅* (b), *M̅*’–*Γ̅*–M̅, and (c) *Y̅*’–*Γ̅*–*Y̅* paths on the (001) plane of the Brillouin zone. The zoomed-in inset represents a clear intersection of surface states from valence and conduction bands, giving the Dirac point. DP represents the Dirac point. Projections of surface states with SOC on the *k*_*x*_–*k*_*y*_ plane at (d) *E* – *E*_F_ = 0.189 eV and (e) *E* – *E*_F_ = 0.350 eV. SS indicates surface states.

Similar kinds of topologically protected crystalline surface states giving a Dirac node along M̅’–*Γ̅*–M̅ as indicated by SS_3_ and SS_4_ are shown in Figure 4b. Other topological surface states marked by SS_5_ and SS_6_ along *Y̅’*–*Γ̅*–*Y̅*. are depicted in Figure 4c, showing a similar pattern. These results indicate that Li_2_CdGe (F4̅3m) exhibits remarkable symmetry-mediated crystalline surface states. Overall, a pair of topological surface states, originating from bulk valence and conduction bands, cross at *Γ̅* to form Dirac-like cones along the *X̅*’–*Γ̅*–*X̅*, *M̅*’–*Γ̅*–*M̅*, and *Y̅*’–*Γ̅*–*Y̅*. directions.

Dirac nodes can also be portrayed as projections of surface states on the Brillouin zone. Therefore, we have demonstrated the surface arcs enclosing the Dirac point by computing the *k*_*x*_ *–k*_*y*_ projection of surface states in different energy level slices. The evolution of these Fermi surface arcs at the (001) plane by varying chemical potentials is indicated in Figure 4d,e. These contours represent different energy slices across the entire surface Brillouin zone, centered at Γ, at *E* – *E*_F_ = 0.189 and 0.350 eV, respectively. The closed circular contour represented in Figure 4d is calculated at *E* – *E*_F_ = 0.189 eV, corresponding to the Dirac point located at *E* – *E*_F_ = 0.189 eV, which is consistent with the bulk band structure presented in Figure 2b. In contrast, the Fermi arc at *E* – *E*_F_ = 0.350 eV [Figure 4e] displays a hexagonal loop surrounding a central circular feature, revealing the evolution of the surface states within this energy window. This hexagonal closed loop, as seen in Figure 4e, stems from contributions of different trivial surface states that are located above the Dirac point at *E* – *E*_F_ = 0.350 eV, which might arise due to hybridization with the bulk band. The surface states SS_2_ to SS_6_ contribute to the arc, as pointed out by the arrow in Figure 4e. Thus, the observed variation in the shape of the Fermi arcs is a direct consequence of the distinct surface state contributions, where the hexagonal arc is linked to trivial states, while the circular arc is attributed to the surface states SS_2_, SS_4_, and SS_6_.

As previously observed in Figure 2e, the electronic band structure of Li_2_CdGe in space group Fm3̅m under PBE-GGA also shows the Dirac point at Γ, originating from the crossing of two degenerate valence and conduction bands. Therefore, we calculated the surface states of Li_2_CdGe in space group Fm3̅m to further verify the Dirac point. Figure S17a-c represents the electronic surface states along the *X̅*’–*Γ*–*X̅*, *M̅*’–*Γ̅*–*M̅*, and *Y̅*’–*Γ̅*–*Y̅* paths on the (001) plane of the Brillouin zone. At Γ, a characteristic crossing of surface states indicates the presence of a Dirac point. The emergence of these gapless surface states at Γ under SOC confirms the nontrivial topological character of Li_2_CdGe in the Fm3̅m phase.

### 3.4 Electronic Structure and Topological Properties of Li_2_CdPb in Space Group Fm3̅m

Out of the five studied compounds, Li_2_CdPb and Li_2_CdSn energetically prefer space group Fm3̅m (No. 225). To demonstrate the topologically protected Dirac nature in the Fm3̅m phase, we considered Li_2_CdPb as a representative compound due to its clearer orbital-projected band characteristics and evident transition from a triple point to a Dirac point near the Fermi level. The PBE-GGA electronic band structures of Li_2_CdPb are given in Figure 5. In the absence of SOC, the electronic band structure shows a crossing of bands in the K−Γ–L direction near the Fermi level at *E* – *E*_F_ = 0.8 eV, as represented in Figure 5a. It can be seen that VBM with two doubly degenerate states touches singly degenerate conduction band minima, forming a triple point. This crossing is shifted to a fourfold band crossing upon the inclusion of SOC, thus forming a Dirac point at Γ, as given in Figure 5b. The accuracy of the results calculated under PBE-GGA was verified by band structure calculation with a hybrid functional under the SOC effect, indicated in Figure 5c. The HSE06 band structure also shows a fourfold degenerate band crossing as observed under PBE-GGA calculations.

**Figure 5:**
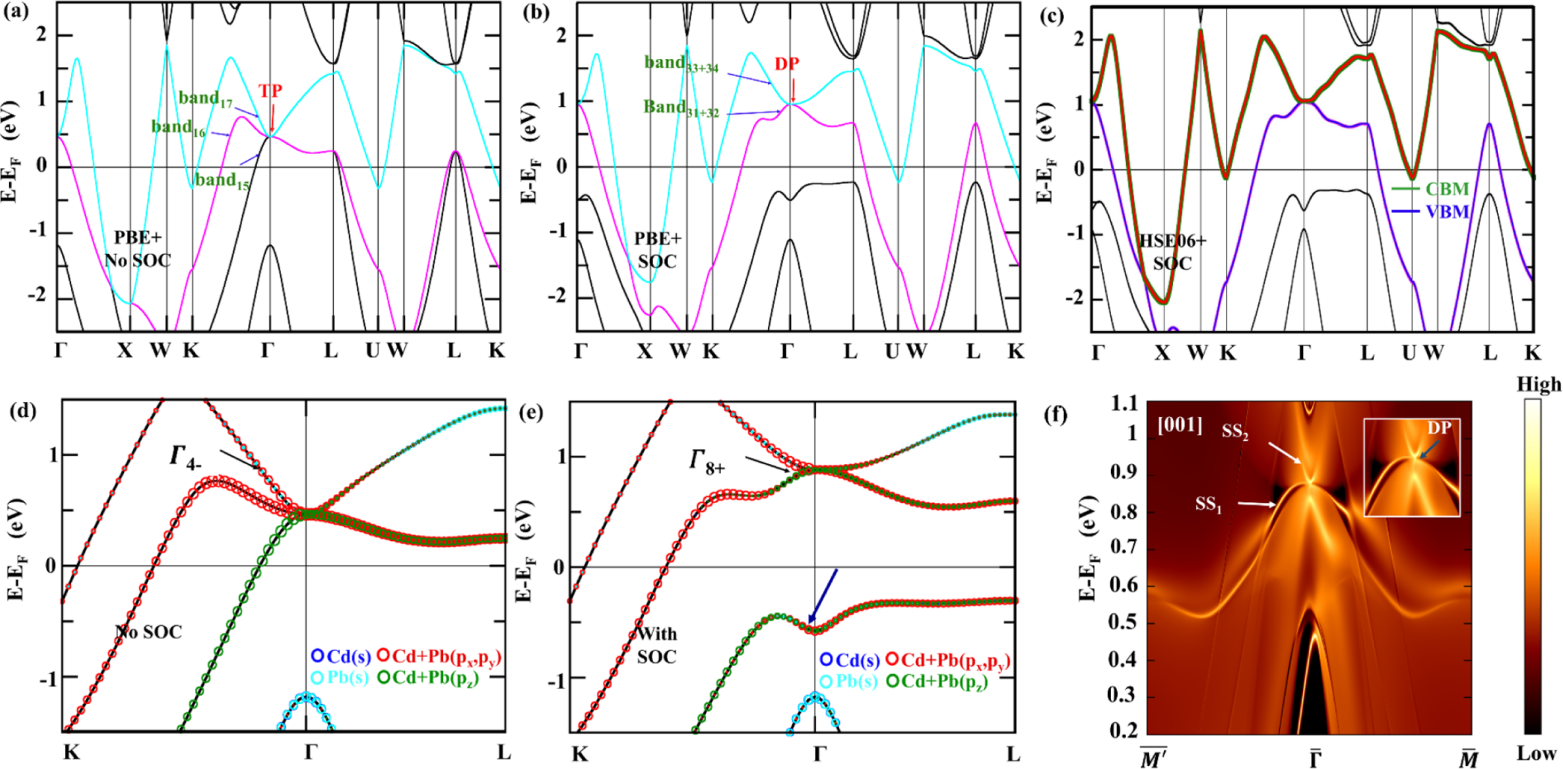
Band structure of Li_2_CdPb in the Fm3̅m (225) phase for (a) without SOC and (b) with SOC under PBE-GGA. Red arrows indicate (a) the triple-point (TP) and (b) the Dirac point (DP). (c) Hybrid functional HSE06 band structure of Li_2_CdPb in space group Fm3̅m (225), showing the conduction band minimum (green) and the valence band maximum (blue). Orbital band projections of Li_2_CdPb (d) without SOC and (e) with SOC in the Fm3̅m (225) phase. Blue and cyan circles represent s orbitals from Cd and Pb, red circles represent p_*x*_ and p_*y*_ states, and green circles represent p_*z*_ states from Cd and Pb, respectively. Black arrows indicated irreps stabilizing TP/DP. Black arrows highlight irreducible representations (irreps) associated with TP/DP protection, and the blue arrow shows the SOC-induced band inversion. (f) Surface band structure for Li_2_CdPb in Fm3̅m (225) showing the topologically protected surface states along *M̅*’–*Γ*–*M̅* on the (001) plane. The zoomed-in inset represents a clear intersection of surface states from valence and conduction bands, giving the Dirac point. SS represents surface states.

Orbital analysis of band crossings along the K−Γ–L direction without and with SOC is shown in Figure 5d and e, respectively. The touching point at Γ involves the contribution of p_*x*_, p_*y*_, and p_*z*_ orbitals from Cd and Pb. After the inclusion of SOC, a clear band inversion is observed, as shown in Figure 5e, where the p_*x*_, p_*y*_, and p_*z*_ orbitals from Cd and Pb undergo splitting and reordering. This leads to the formation of a curved, inverted-warped band that lies energetically 0.7 eV below the Fermi level, signifying a topological phase transition in Li_2_CdPb. This band inversion is highlighted by the blue arrow in Figure 5e. Upon inclusion of SOC, the irreducible representation at the Γ point changes from Γ_4–_ to Γ_8+_, further confirming the topological phase transition.

Surface states obtained by generating maximally localized Wannier functions under PBE-GGA with SOC further confirmed the Dirac-based topological nature of Li_2_CdPb (space group Fm3̅m). To show the Dirac point, we calculated surface states along *X̅*’–*Γ̅*–*X*, *M̅*’–*Γ*–*M*, and *Y̅*’–*Γ̅*–*Y̅* paths on the (001) plane of the Brillouin zone. For comprehensibility, we have shown a clear representation of surface spectra along *M̅*’–*Γ̅*–*M* in Figure 5f where two topological surface states marked by SS_1_ (originating from the valence band) and SS_2_ (coming from the conduction band) at *E* – *E*_F_ = 0.889 eV form a Dirac cone along the path *M̅*’–*Γ̅*–*M̅* on the (001) surface of the Brillouin zone. The inset in Figure 5f shows a zoomed-in view of the Dirac point. The surface states for Li_2_CdPb along *X̅*’–*Γ*–*X̅*, and *Y̅*’–*Γ̅*–*Y̅* paths are given in Figure S18, where surface-state spectra represent the Dirac cone at Γ formed by topological surface states SS_3_ and SS_4_ (along *X̅*’–*Γ̅*–*X̅*) and SS_5_ and SS_6_ (along *Y̅*’–*Γ̅*–*Y̅*).

The PBE-GGA band structures for Li_2_CdPb, Li_2_CdSn, Li_2_ZnGe, Li_2_ZnPb, and Li_2_ZnSn in both structural phases are consolidated in Figures S2–S6, while band structures under HSE06 are shown in Figures S7–S11. To highlight the nontrivial nature of these compounds, the orbital band projections are provided in Figures S12–S16. These orbital band projections indicate the natural reordering of s and p orbitals and SOC-induced band inversion (curved-warped band) in Li_2_CdPb, Li_2_CdSn, and Li_2_ZnPb in the F4̅3m phase and in Li_2_CdPb, Li_2_CdSn, Li_2_ZnGe, Li_2_ZnPb, and Li_2_ZnSn in the Fm3̅m phase.

### 3.5 Parity Analysis and Wannier Charge Center Evolution

We further confirm the nontrivial nature of Li_2_YZ materials through the calculation of *Z*_2_ topological invariant by two methods: (i) parity analysis based on the Fu–Kane model^91^ applicable to the centrosymmetric phase (Fm3̅m space group) and (ii) evolution of Wannier charge centers (WCCs) by using WannierTools^89^ for both centrosymmetric and noncentrosymmetric cases (F4̅3m phase). The *Z*_2_ topological invariant for bulk materials is characterized by four indices (*v*_0_; *v*_1_, *v*_2_, and *v*_3_). A strong topological phase is indicated when the topological index ν_0_ = 1, while ν_0_ = 0 corresponds to either a weak topological or a trivial phase. A nonzero value of any of the three indices (ν_1_, ν_2_, ν_3_) when ν_0_ = 0 indicates a weak topological phase, while a zero value of all four indices represents a trivial phase.

The parity analysis for Li_2_YZ compounds is summarized in Tables S3–S8. At Γ, three occupied bands (*n* = 34, 32, and 30) exhibit odd parity corresponding to p*-*type states, whereas the lower band (*n* = 28) has even parity corresponding to s*-*type states. For all materials, the shift in parity sign from odd to even at Γ is consistent with the reversal in the order of the p-type and s-type states. For Li_2_CdGe, this parity flip is presented in Figure 2h. In the case of Li_2_CdPb, Li_2_CdSn, Li_2_ZnGe, Li_2_ZnPb, and Li_2_ZnSn, this parity reversal has been demonstrated in Figures S12d, S13d, S14d, S15d, and S16d, respectively. Thus, in all cases, the product of parity eigenvalues over eight time reversal invariant momenta (TRIM) points yields a strong topological index of ν_0_ = 1.

The evolution of WCCs was used to verify the *Z*_2_ topological invariant for the representative materials, Li_2_CdGe in space group F4̅3m and Li_2_CdPb in space group Fm3̅m. The evolution of WCCs determined *Z*_2_ for the representative materials in six time reversal invariant planes, i.e., *k*_1_ = 0, 0.5; *k*_2_ = 0, 0.5; and *k*_3_ = 0, 0.5. The topological character is analyzed by counting the number of crossings between WCC curves and the horizontal reference line. An even number of crossings between the horizontal reference line and WCC curves gives *Z*_2_ = 0, whereas an odd number of crossings yields *Z*_2_ = 1, indicating a nontrivial nature.

Figure 6 represents the evolution of WCCs for Li_2_CdGe in phase F4̅3m. In Figure 6a,c,e, the WCC curve crosses the reference line an odd number of times, indicating *Z*_2_ = 1, while in Figure 6b,d,f, the WCC curve crosses the reference line an even number of times, indicating *Z*_2_ = 0. Thus, we have *Z*_2_(*k*_1_ = 0) = 1; *Z*_2_(*k*_1_ = 0.5) = 0; *Z*_2_(*k*_2_ = 0) = 1; *Z*_2_(*k*_2_ = 0.5) = 0; *Z*_2_(*k*_3_ = 0) = 1; and *Z*_2_(*k*_3_ = 0.5) = 0. This results in an overall *Z*_2_ invariant of (1;000), which confirms the nontrivial nature of Li_2_CdGe (F4̅3m), further supported by the presence of a Dirac point in the calculated surface-state spectra, as shown in Figure 4. Also, the evolution of WCCs for the other representative material Li_2_CdPb in space group Fm3̅m is provided in Figure S19. By using the standard rule of counting WCC crossings with the reference line indicated, we obtained *Z*_2_(*k*_1_ = 0) = 0; *Z*_2_(*k*_1_ = 0.5) = 0; *Z*_2_(*k*_2_ = 0) = 1; *Z*_2_(*k*_2_ = 0.5) = 0; *Z*_2_(*k*_3_ = 0) = 0; and *Z*_2_(*k*_3_ = 0.5) = 0. Thus, the overall *Z*_2_ topological index is (1;000). Hence, the two methods are consistent in demonstrating the nontrivial topologies in Li_2_CdPb.

**Figure 6:**
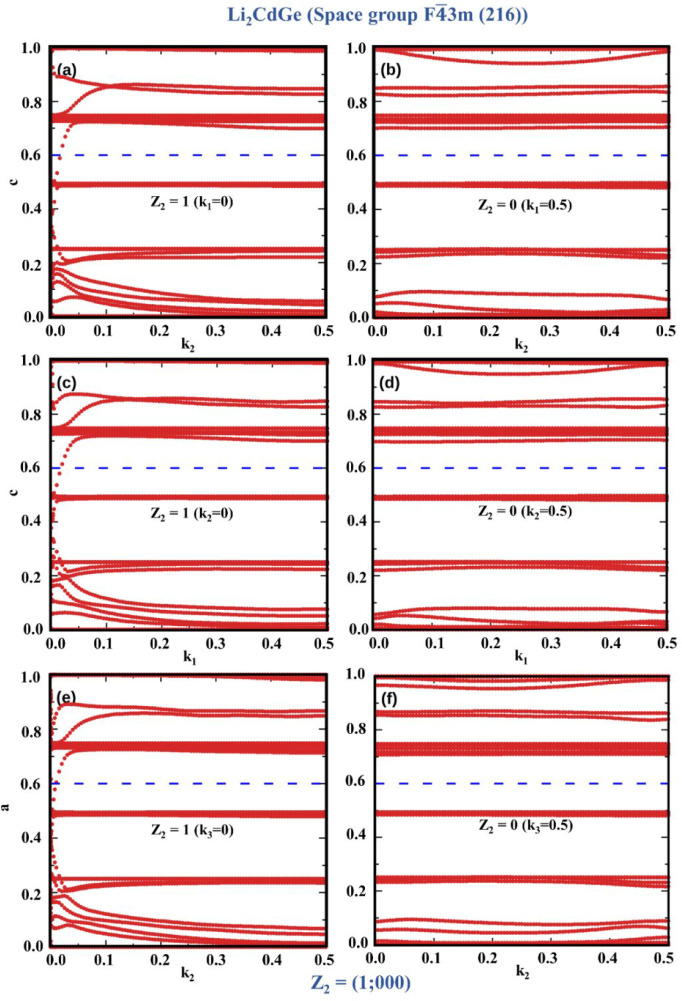
Evolution of Wannier charge centers (WCCs) for Li_2_CdGe in space group F4̅3m (216), calculated for the six time-reversal-invariant planes in the Brillouin zone: (a) *k*_1_ = 0, (b) *k*_1_ = 0.5, (c) *k*_2_ = 0, (d) *k*_2_ = 0.5, (e) *k*_3_ = 0, and (f) *k*_3_ = 0.5. The calculated *Z*_2_ invariants for each plane are indicated in the corresponding panels. The blue line represents the reference line.

### 3.6 Topological Analysis of Li_2_YZ Compounds

We further assessed the topological character of six Li_2_YZ compounds by using the SymTopo^87^ tool via symmetry-indicator analysis of irreducible representations (irreps) of occupied bands, considering high-symmetry momenta. Table S9 summarizes the topological or trivial phase in Li_2_YZ compounds, such as high-symmetry-point semimetal (HSPSM), normal insulator (NI), or semimetal (SM) in both space groups, with and without considering SOC obtained from SymTopo analysis. The table demonstrates the *k*-points where symmetry-enforced degeneracies exist (e.g., Γ or X), band indices at degenerate points, and irreps at those high-symmetry points. The dimension equals the degeneracy reported in parentheses next to the band indices in Table S9.

All Li_2_YZ compounds are topologically nontrivial, specifically high-symmetry-point semimetals, both without and with SOC in space group Fm3̅m (225). Without SOC, bands are threefold degenerate at the Γ point and twofold degenerate at the X point with irreps Γ_4‑_ and X_5‑_, respectively. With the inclusion of SOC, bands at the Γ point become fourfold degenerate, giving a Dirac-type crossing with irreps Γ_8+_, yielding a HSPSM classification.

For space group F4̅3m, Li_2_CdGe, Li_2_CdPb, and Li_2_ZnPb are topological semimetals, particularly high-symmetry-point semimetals, with threefold degeneracy without SOC and fourfold degeneracy with SOC at Γ. Li_2_ZnGe and Li_2_ZnSn are identified to be trivial materials, while Li_2_CdSn in space group F4̅3m undergoes a topological phase transition from a trivial material to a high-symmetry-point semimetal under SOC.

The SymTopo code predicts the topological properties by using the symmetry representation of crystalline materials. A few theoretical studies^101–104^ relating irreducible representations (irreps) at high-symmetry momenta to topological invariants and symmetry-enforced band nodes serve as the foundation for this technique. The code initially checks the number of electrons per unit cell for a nonmagnetic crystal; structures with an odd count are called typical metals. If not, the crystal is standardized to a primitive-cell convention (consistent symmetry operators and high-symmetry points or HSPs), and the space group is determined. The standardized cell is next subjected to density-functional calculations with and without spin–orbit coupling (SOC) to derive Bloch wave functions and eigenvalues at every HSP.

After determining high-symmetry point irreps, SymTopo analyzes if any high-symmetry point has a direct band gap below the threshold value ≈2 meV. The output generated from SymTopo includes *k*-point (for instance, Γ), band indices (for instance, bands 31–34), irreps (for instance, Γ_8_), and dimensionality of the multiplet (for instance, 4D). For the case where valence band maxima and conduction band minima belong to the same symmetry multiplet, the system is classified as a high-symmetry-point semimetal (HSPSM). In the case of absence of high-symmetry point degeneracy, the SymTopo code breaks high-symmetry point irreps into the line irreps to test the compatibility relations along each linked high-symmetry line/surface. In the case of any violation of these relations, the system is considered a high-symmetry-line semimetal (HSLSM), where band crossings lie along the symmetry-enforced lines.

Symmetry-based indicators are computed as integer combinations of high-symmetry point irreps’ multiplicities. Without SOC, a nonzero indicator identifies a generic-momentum semimetal, where nodes lie away from high-symmetry points or lines. When SOC is taken into account, a nonzero indicator signals a topological insulator (TI) or a topological crystalline insulator (TCI), by considering space group-specific mapping. While a zero indicator (or for the systems where no indicators are defined for the space groups) does not guarantee triviality, it implies that topology is not detected by symmetry eigenvalue analysis and must be resolved through complementary probes.

## 4 Conclusion

We demonstrated a structural, electronic, and topological analysis of Li_2_YZ in two cubic phases, F4̅3m and Fm3̅m. To illustrate the electronic structure properties based on orbital projection analysis and surface states in both F4̅3m and Fm3̅m phases, we considered Li_2_CdGe as a representative case. No imaginary frequencies were observed in the phonon spectra of Li_2_CdGe in both phases, implying dynamic stability. We further analyzed a fourfold Dirac semimetallic nature of Li_2_CdGe, a full-Heusler material, in two space groups F4̅3m and Fm3̅m. Results indicate the emergence of distinct surface states on the (001) surface of the Brillouin zone in Li_2_CdGe in both crystal structures. These surface states are robust due to symmetry-protected band degeneracies. For a comprehensive investigation on space group F4̅3m, Li_2_CdGe was chosen, while for examining the electronic and topological properties in the Fm3̅m phase, Li_2_CdPb was taken into account. We further predicted nontrivial behavior in all Li_2_YZ materials in the Fm3̅m space group, while in four Li_2_YZ materials (Li_2_CdGe, Li_2_CdPb, Li_2_CdSn, and Li_2_ZnPb) in the F4̅3m space group, based on their electronic band structure and symmetry-based analysis.

For completeness, we note that the Wannier charge center (WCC)/Wilson loop procedure can be applied consistently to both centrosymmetric and noncentrosymmetric cases and agrees with the Fu–Kane parity analysis whenever inversion symmetry is present.

Our findings suggest that this group of full-Heusler compounds possesses unique characteristics and is promising for use in electronic and topological applications. The results indicate that Li_2_YZ compounds possess nontrivial topological features that have great potential for theoretical and experimental study in materials science and condensed matter.

## Supplementary Material



## Data Availability

The data that support the findings of this study are available within the article and its Supporting Information.

## Notes

Bond lengths in Li_2_CdGe in the F4̅3m and Fm3̅m phases; phonon dispersion spectra of Li_2_YZ compounds; surface states of Li_2_CdGe and orbital band projections (along K−Γ–L) in the Fm3̅m phase; surface states of Li_2_CdPb; electronic band structures of Li_2_YZ under PBE-GGA and HSE06 in Fm3̅m and F4̅3m phases; orbital band projections of Li_2_YZ under PBE-GGA; Li_2_YZ parity analysis of all occupied bands in Fm3̅m phase and evolution of Wannier charge centers (WCCs) of Li_2_CdPb in the Fm3̅m phase; and the table of output from SymTopo calculations (PDF)
