# Supplementary material for: Topological Dirac Semimetallic Phase in Heusler-Type Li2YZ (Y = Zn or Cd and Z = Ge, Sn, or Pb) Compounds: A First-Principles Investigation
Source: ACS Omega. 2025 Nov 19;10(47):56973–86. doi: 10.1021/acsomega.5c01191 (PMC12676365; doi:10.1021/acsomega.5c01191)
Supplement: Supplementary file 1 [file ao5c01191_si_001.pdf]

Supporting Information for

Topological Dirac semimetallic phase in Heusler-type Li_2YZ (Y = Zn or Cd; and Z = Ge, Sn, or Pb) compounds: A first-principles investigation

Fareeha Waheed[1], Ina Marie R. Verzola[1], Sreeparvathy Puthiya Covilakam[1,2,3], Rovi Angelo B. Villaos[1], Ancieto B. Maghirang[1,2,3], Zhi-Quan Huang[1], and Feng-Chuan Chuang[1,2,3,4,*]

[1]*Department of Physics, National Sun Yat-Sen University, Kaohsiung 80424, Taiwan*

[2]*Physics Division, National Center for Theoretical Sciences, Taipei 10617, Taiwan*

[3]*Center for Theoretical and Computational Physics, National Sun Yat-Sen University, Kaohsiung 80424, Taiwan*

[4]*Department of Physics, National Tsing Hua University, Hsinchu 30013, Taiwan*

*Corresponding Author: fchuang@mail.nsysu.edu.tw

Postal Address: 70 Lienhai Rd., Kaohsiung 80424, Taiwan.

Telephone: +886-7-5253733

1. Structure and Stability of Li_2YZ (Y = Zn or Cd; and Z = Ge, Sn, or Pb) Compounds.

1.1. Structural comparison of two space groups, $F\bar{4}3m$ (No. 216) and $Fm\bar{3}m$ (No. 225) based on the primitive unit cell of Li_2CdGe.

Table S1. The calculated atomic bond lengths in the Li_2CdGe primitive unit cell in the two space groups $F\bar{4}3m$ and $Fm\bar{3}m$.

Space group $F\bar{4}3m$ (No. 216)		Space group $Fm\bar{3}m$ (No. 225)	
**Bond**	**Distance (Å)**	**Bond**	**Distance (Å)**
**Li_1-Cd_1**	3.25 Å	**Li_1-Cd_1**	2.79 Å
**Li_2-Cd_1**	2.81 Å	**Li_2-Cd_1**	2.79Å
**Li_1-Ge_1**	2.81 Å	**Li_1-Ge_1**	2.79 Å
**Li_2-Ge_1**	3.25 Å	**Li_2-Ge_1**	2.79 Å
**Cd_1-Ge_1**	2.81 Å	**Ge_1-Cd_1**	3.22 Å
**Cd_1-Ge_1**	5.39 Å	**Cd_1-Ge_1**	5.58 Å

1.2. Comparison of the total ground-state energies of the cubic primitive unit cell in space group $F\bar{4}3m$ (No. 216) and $Fm\bar{3}m$ (No. 225).

Table S2. The total ground state energy of Li_2YZ (Y = Zn or Cd; and Z = Ge, Sn, or Pb) in electronvolts per formula unit (eV/f.u.) under PBE-GGA with the inclusion of SOC for the two possible structural phases. The energy values in red color refer to the energetically preferred structure.

Li_2YZ Materials	Total Ground State Energy (eV/f.u.)	
	Space group $F\bar{4}3m$ (No. 216)	Space group $Fm\bar{3}m$ (No. 225)
Li_2CdGe	**-10.301**	-10.179
Li_2CdSn	-9.810	**-9.811**
Li_2CdPb	-9.696	**-9.858**
Li_2ZnGe	**-10.782**	-10.418
Li_2ZnSn	**-10.133**	-9.923
Li_2ZnPb	**-9.926**	-9.925

1.3. Phonon dispersion curves of Li_2YZ (Y = Zn or Cd; and Z = Ge, Sn, or Pb) compounds in their energetically preferred phases.

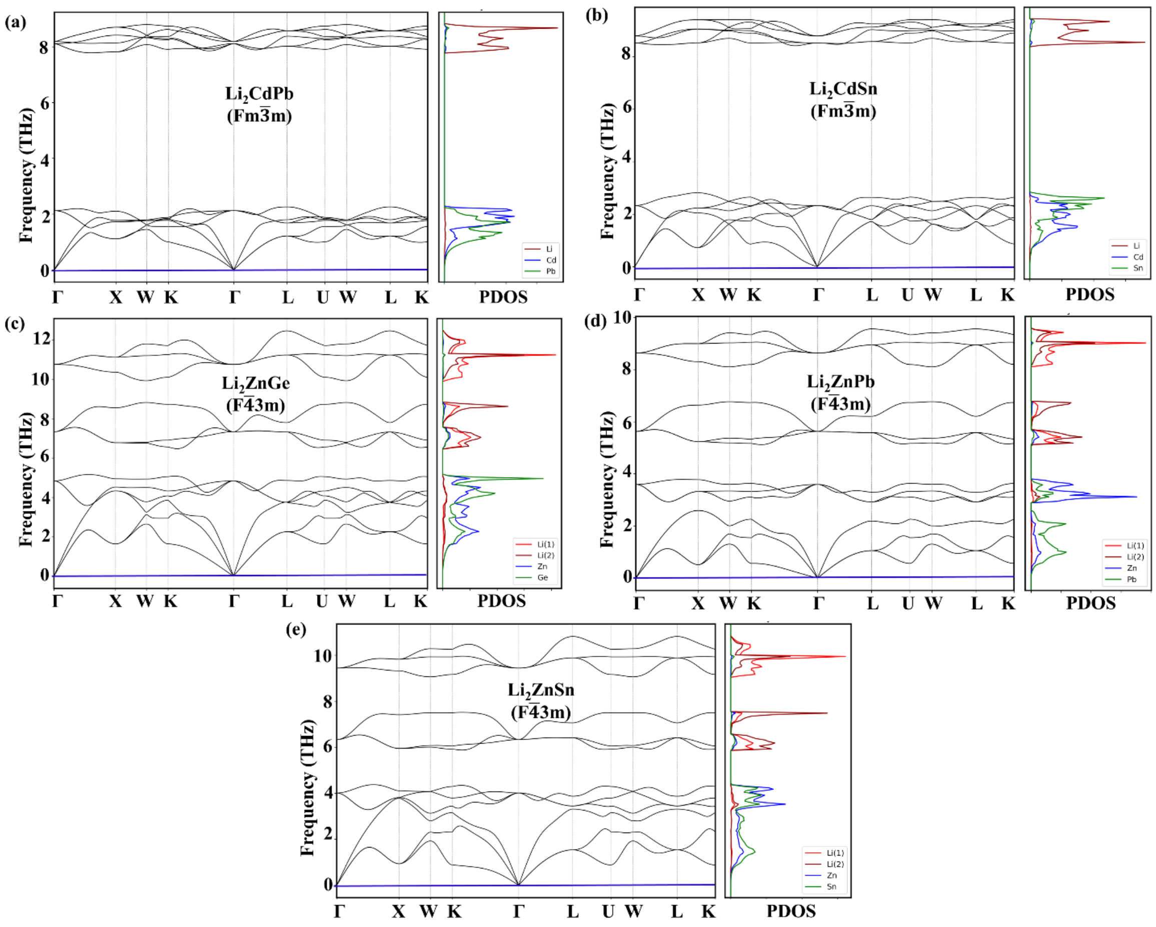

FIG. S1. The phonon dispersion curves and partial phonon density of states (PDOS) of Li_2YZ compounds (a) Li_2CdPb and (b) Li_2CdSn in space group $Fm\bar{3}m$, and (c) Li_2ZnGe, (d) Li_2ZnPb, and (e) Li_2ZnSn in space group $F\bar{4}3m$.

2. Electronic Structure and Topological Properties of Li_2YZ.

2.1. Electronic band structures of Li_2YZ under PBE-GGA and HSE06 in $F\bar{4}3m$ and $Fm\bar{3}m$ phases.

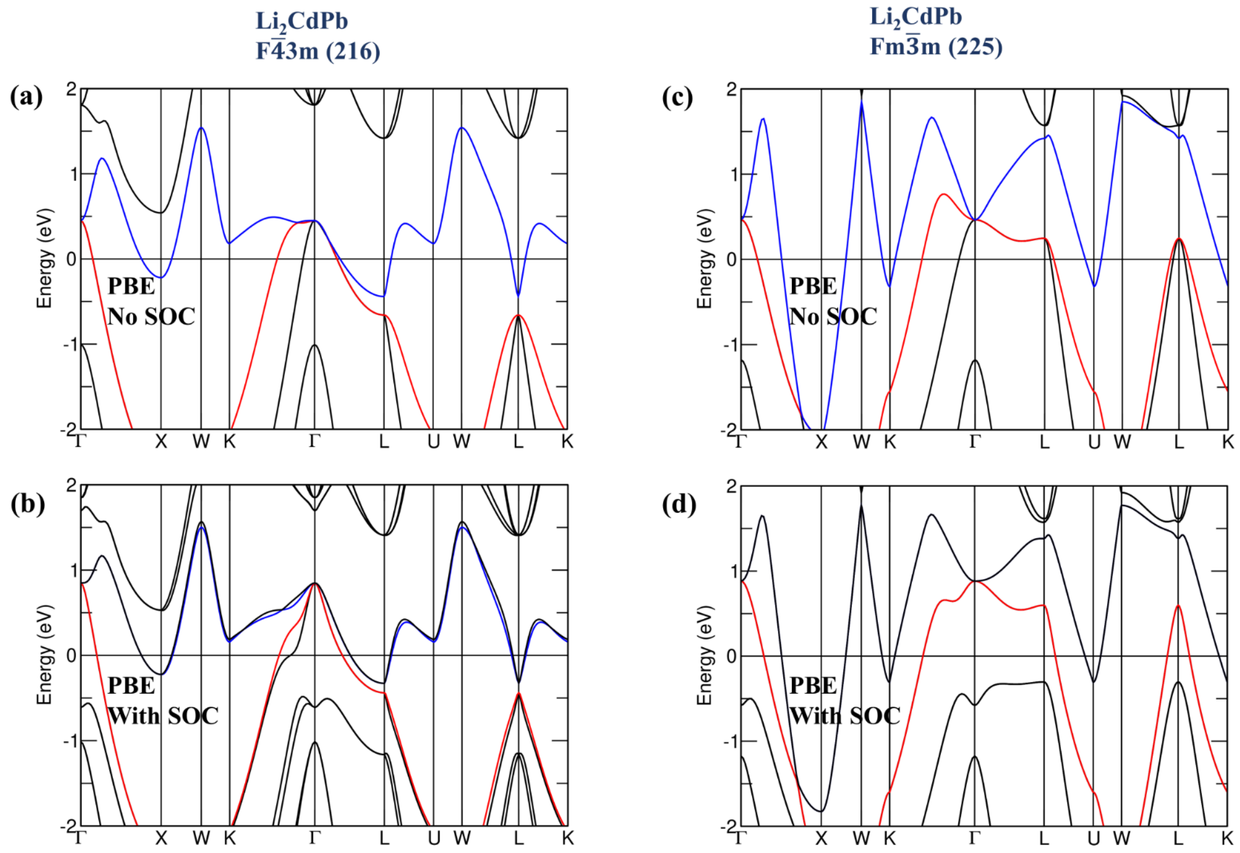

FIG. S2. Band structures of the bulk Li_2CdPb under PBE-GGA. Band structure of Li_2CdPb (a) without SOC and (b) with SOC in space group $F\bar{4}3m$. Band structure of Li_2CdPb (c) without SOC and (d) with SOC in space group $Fm\bar{3}m$.

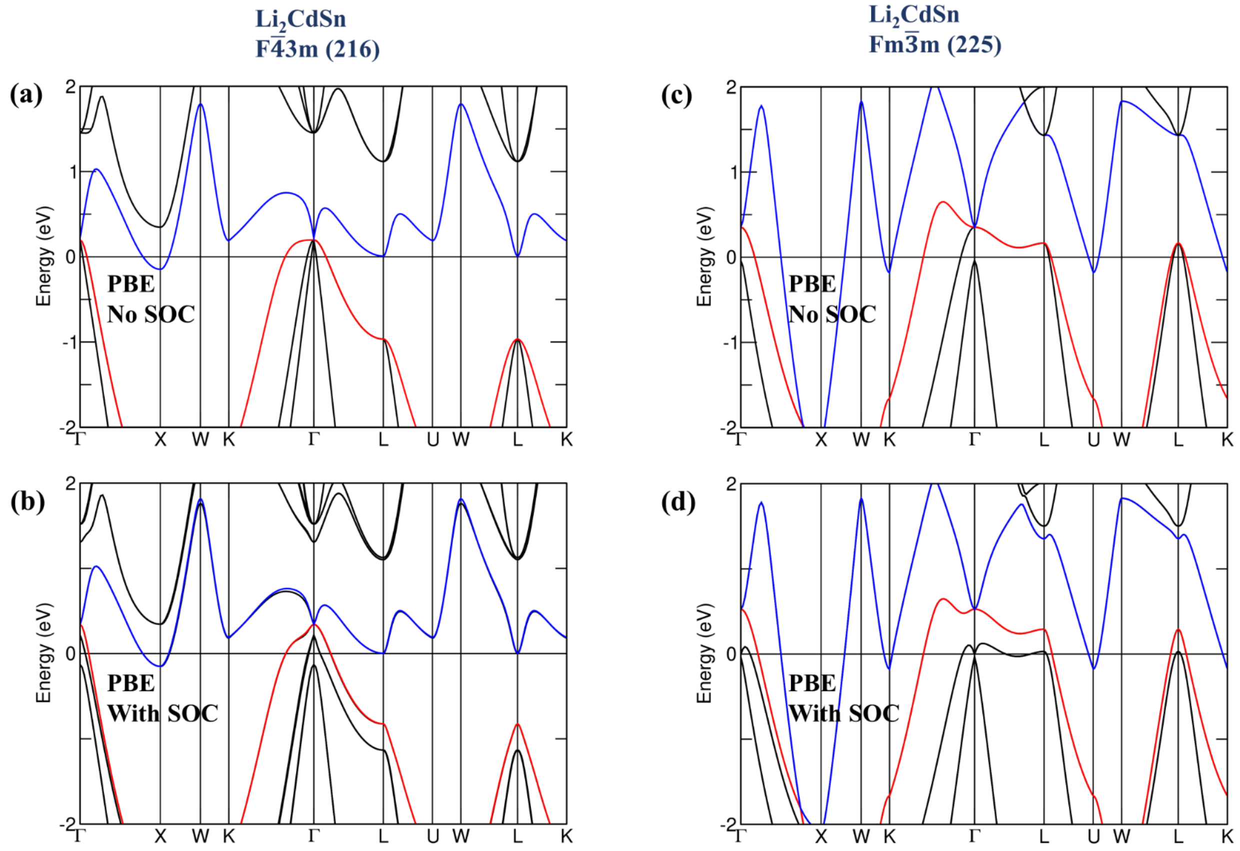

FIG. S3. Band structures of the bulk Li_2CdSn under PBE-GGA. Band structure of Li_2CdSn (a) without SOC and (b) with SOC in space group $F\overline{4}3m$. Band structure of Li_2CdSn (c) without SOC and (d) with SOC in space group $Fm\overline{3}m$.

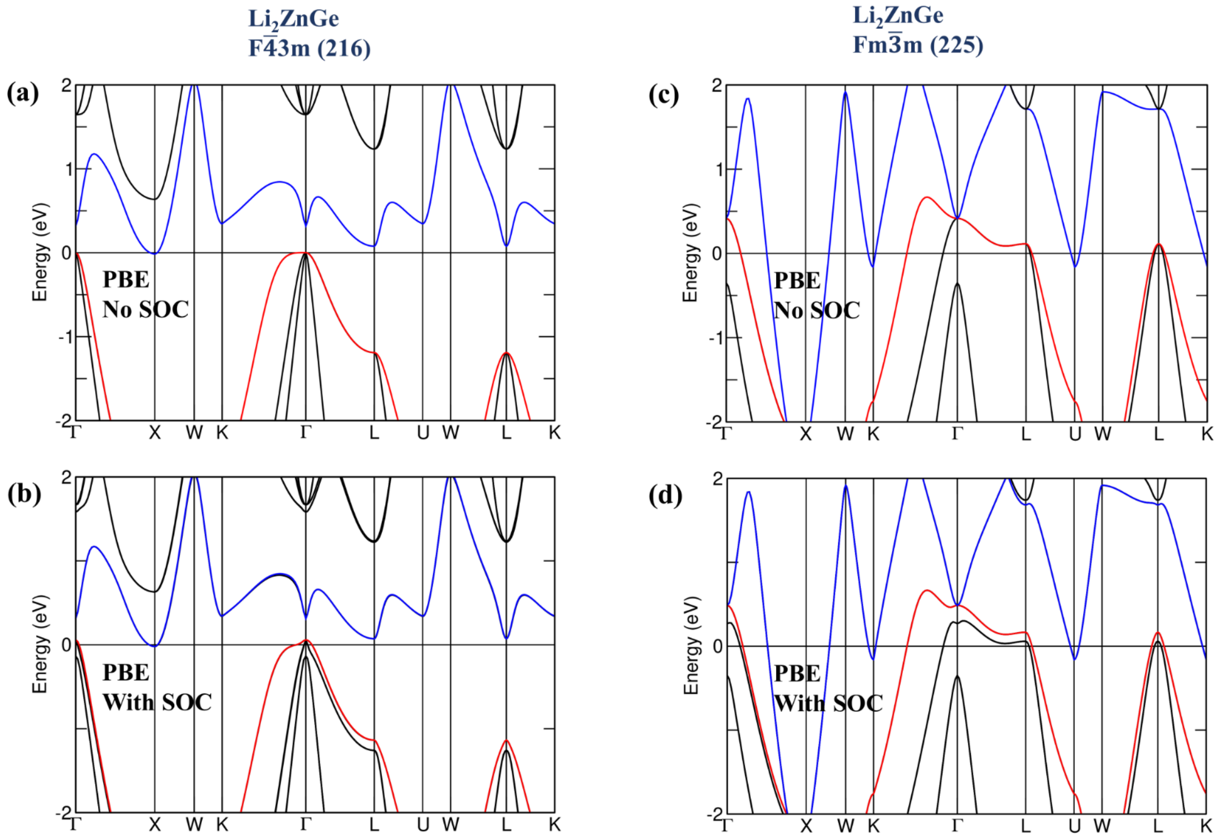

FIG. S4. Band structures of the bulk Li_2ZnGe under PBE-GGA. Band structure of Li_2ZnGe (a) without SOC and (b) with SOC in space group F$\overline{4}$3m. Band structure of Li_2ZnGe (c) without SOC and (d) with SOC in space group Fm$\overline{3}$m.

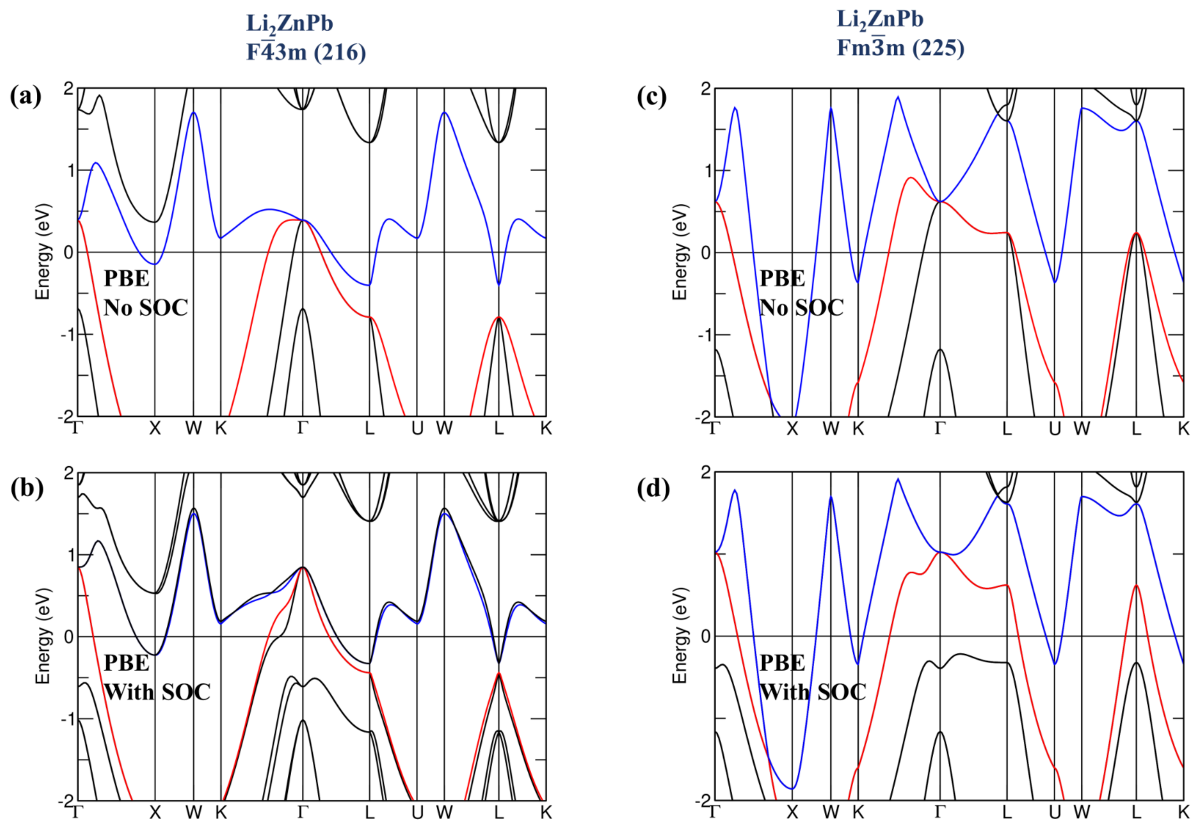

FIG. S5. Band structures of the bulk Li_2ZnPb under PBE-GGA. Band structure of Li_2ZnPb (a) without SOC and (b) with SOC in space group F$\bar{4}$3m. Band structure of Li_2ZnPb (c) without SOC and (d) with SOC in space group Fm$\bar{3}$m.

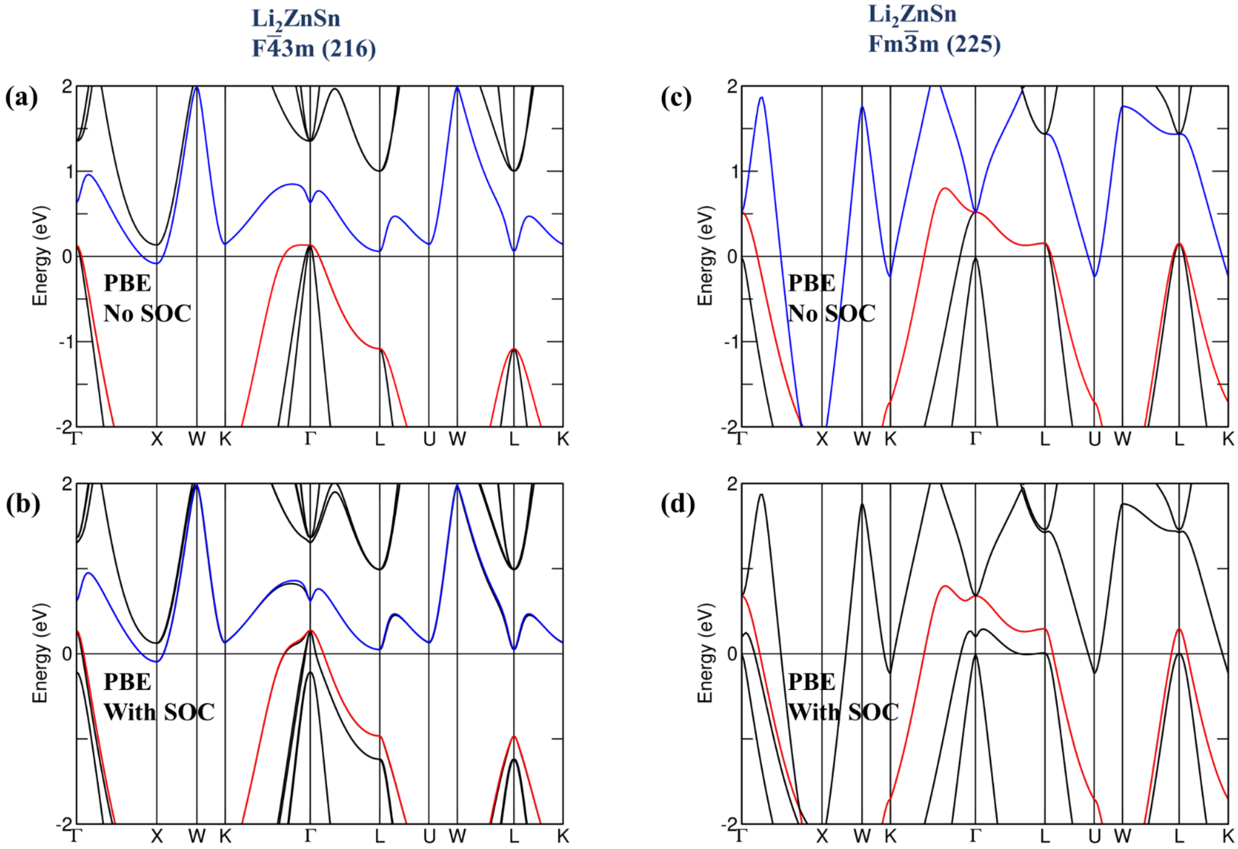

FIG. S6. Band structures of the bulk Li_2ZnSn under PBE-GGA. Band structure of Li_2ZnSn (a) without SOC and (b) with SOC in space group $F\bar{4}3m$. Band structure of Li_2ZnSn (c) without SOC and (d) with SOC in space group $Fm\bar{3}m$.

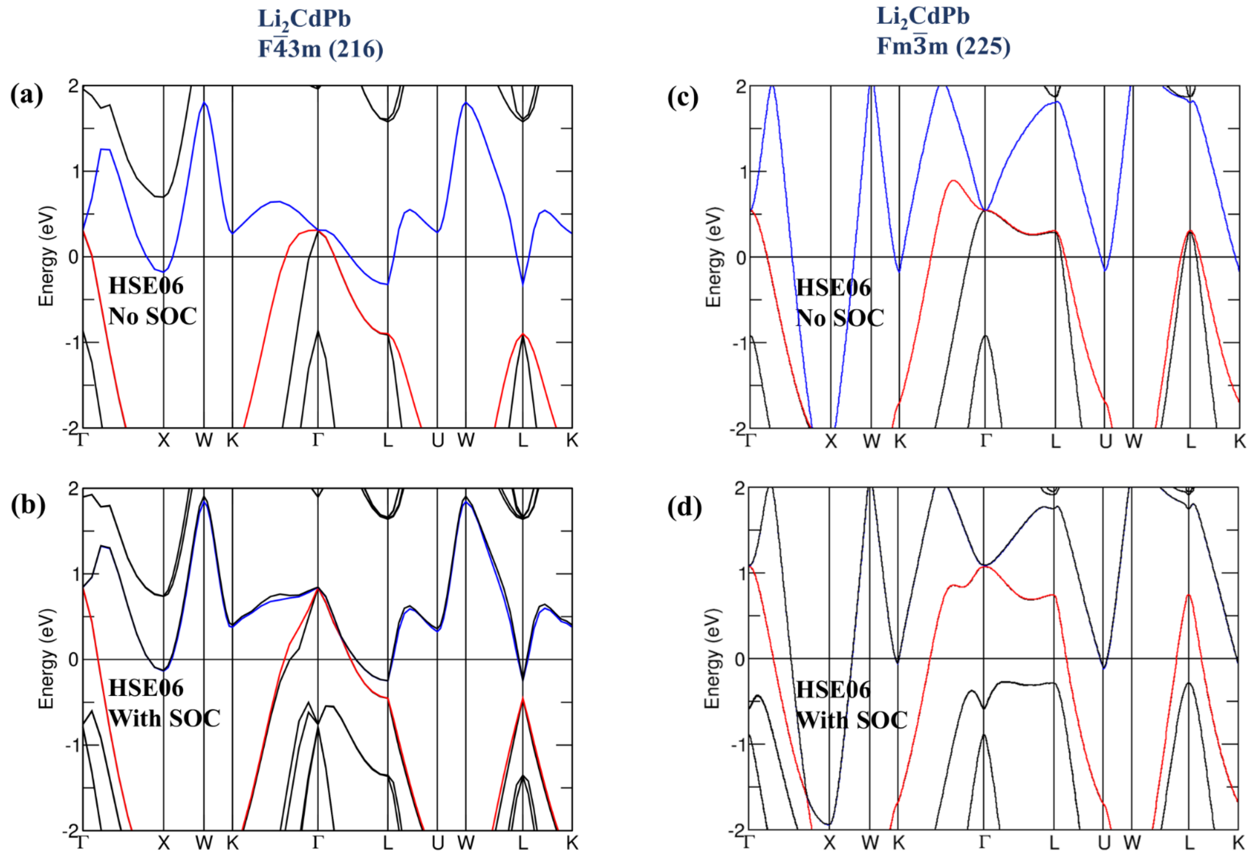

FIG. S7. Band structures of the bulk Li_2CdPb under HSE06. Band structure of Li_2CdPb (a) without SOC and (b) with SOC in space group $F\bar{4}3m$. Band structure of Li_2CdPb (c) without SOC and (d) with SOC in space group $Fm\bar{3}m$.

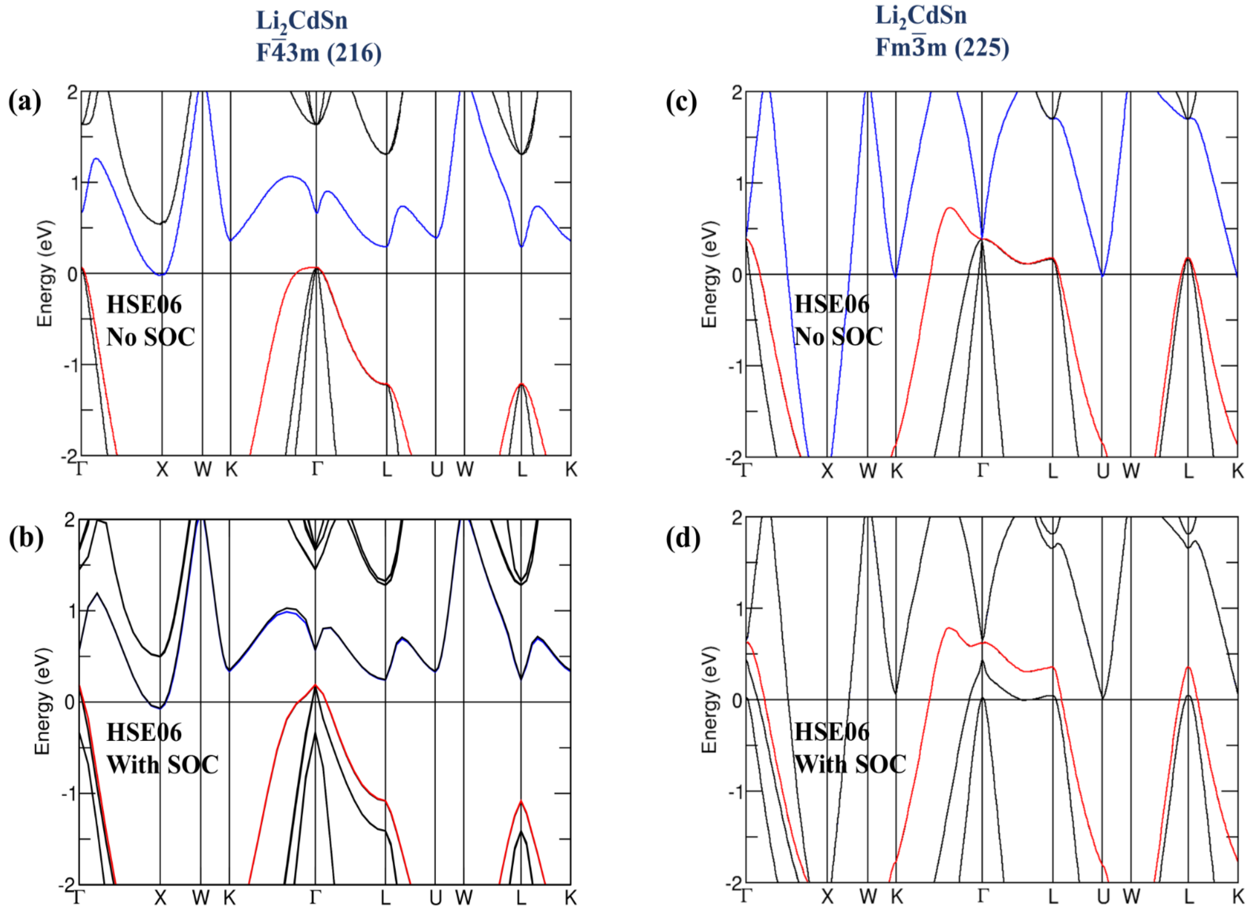

FIG. S8. Band structures of the bulk Li_2CdSn under HSE06. Band structure of Li_2CdSn (a) without SOC and (b) with SOC in space group $F\bar{4}3m$. Band structure of Li_2CdSn (c) without SOC and (d) with SOC in space group $Fm\bar{3}m$.

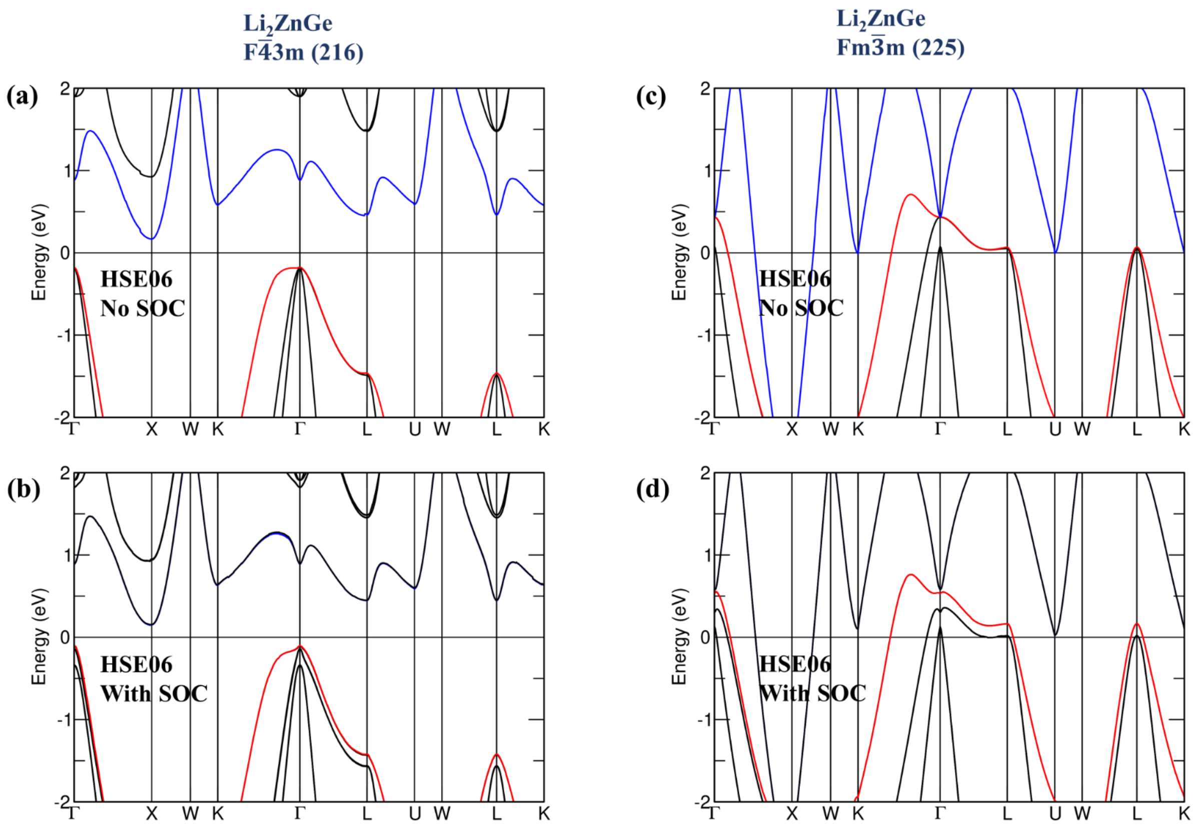

FIG. S9. Band structures of the bulk Li_2ZnGe under HSE06. Band structure of Li_2ZnGe (a) without SOC and (b) with SOC in space group $F\overline{4}3m$. Band structure of Li_2ZnGe (c) without SOC and (d) with SOC in space group $Fm\overline{3}m$.

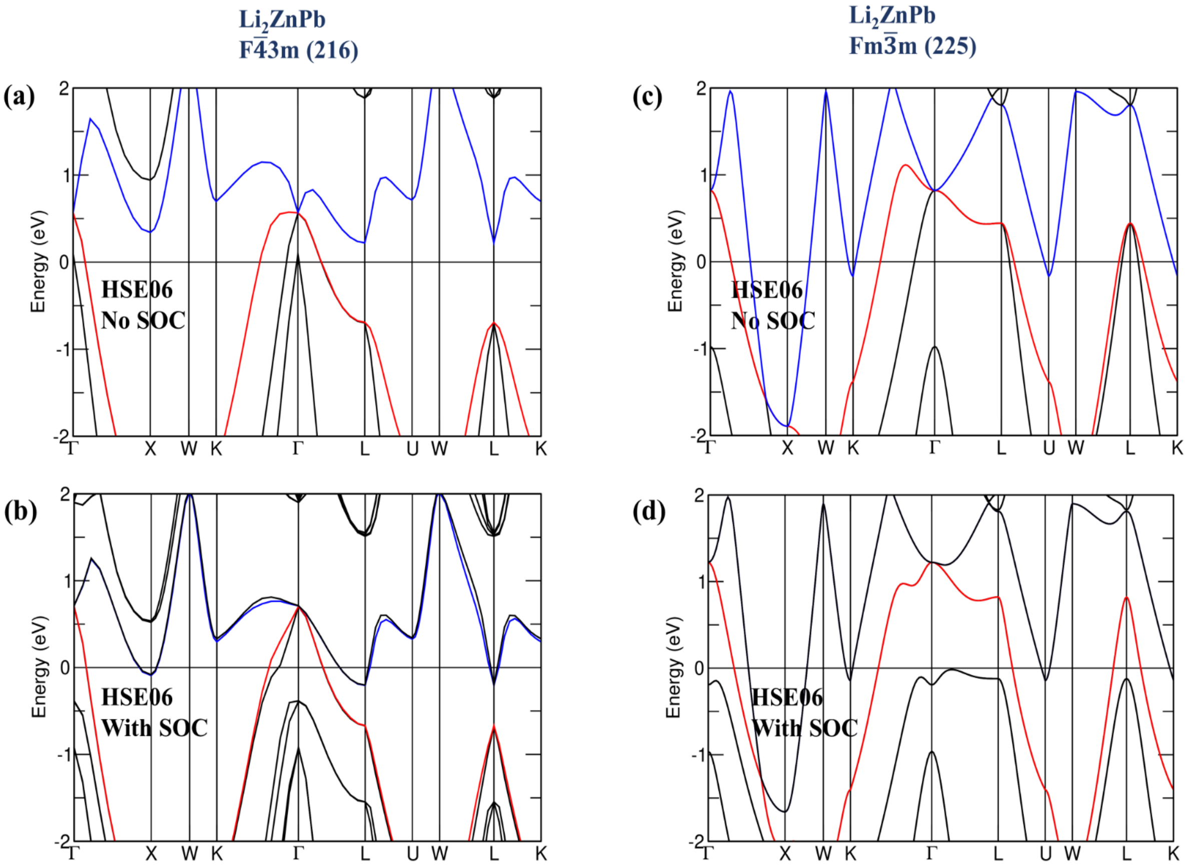

FIG. S10. Band structures of the bulk Li_2ZnPb under HSE06. Band structure of Li_2ZnPb (a) without SOC and (b) with SOC in space group $F\bar{4}3m$. Band structure of Li_2ZnPb (c) without SOC and (d) with SOC in space group $Fm\bar{3}m$.

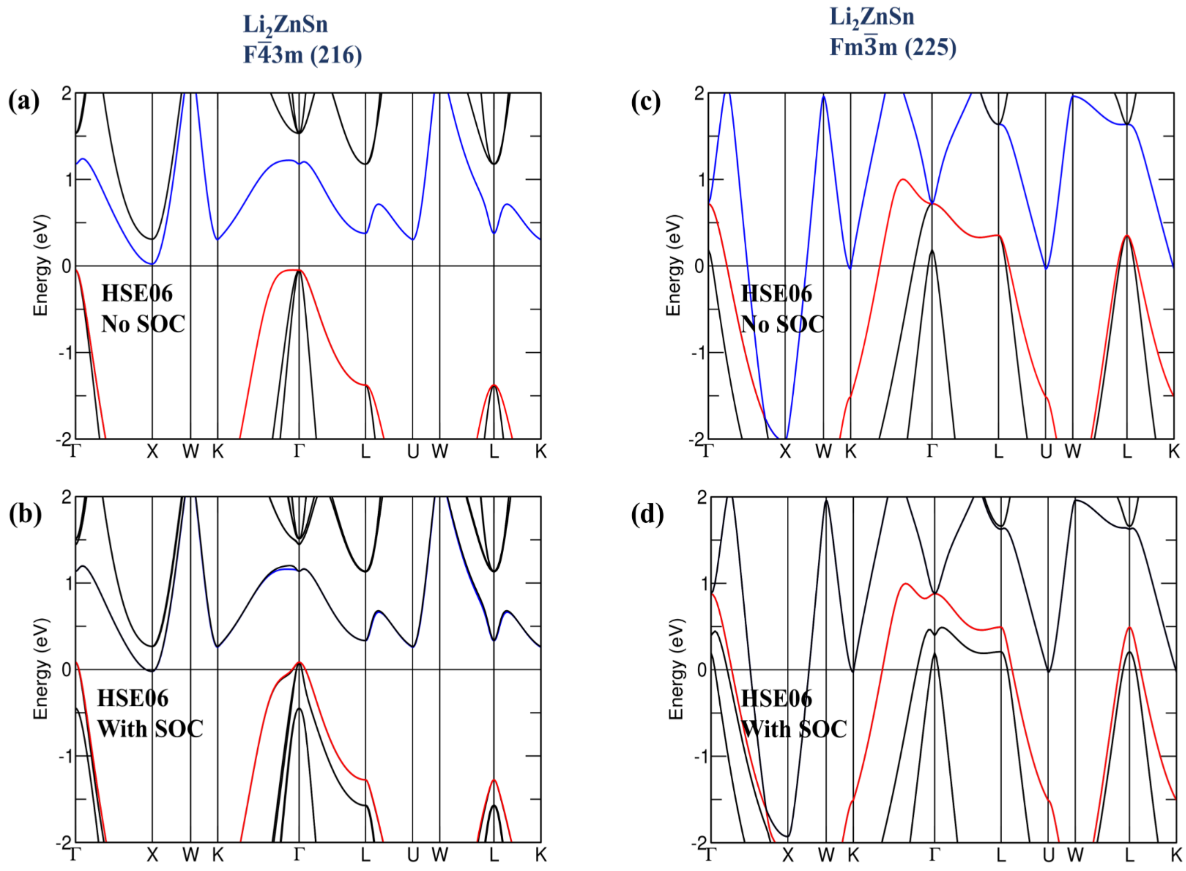

FIG. S11. Band structures of the bulk Li_2ZnSn under HSE06. Band structure of Li_2ZnSn (a) without SOC and (b) with SOC in space group $F\bar{4}3m$. Band structure of Li_2ZnSn (c) without SOC and (d) with SOC in space group $Fm\bar{3}m$.

2.2. Orbital band projections of Li_2YZ under PBE-GGA in $F\bar{4}3m$ and $Fm\bar{3}m$ phases.

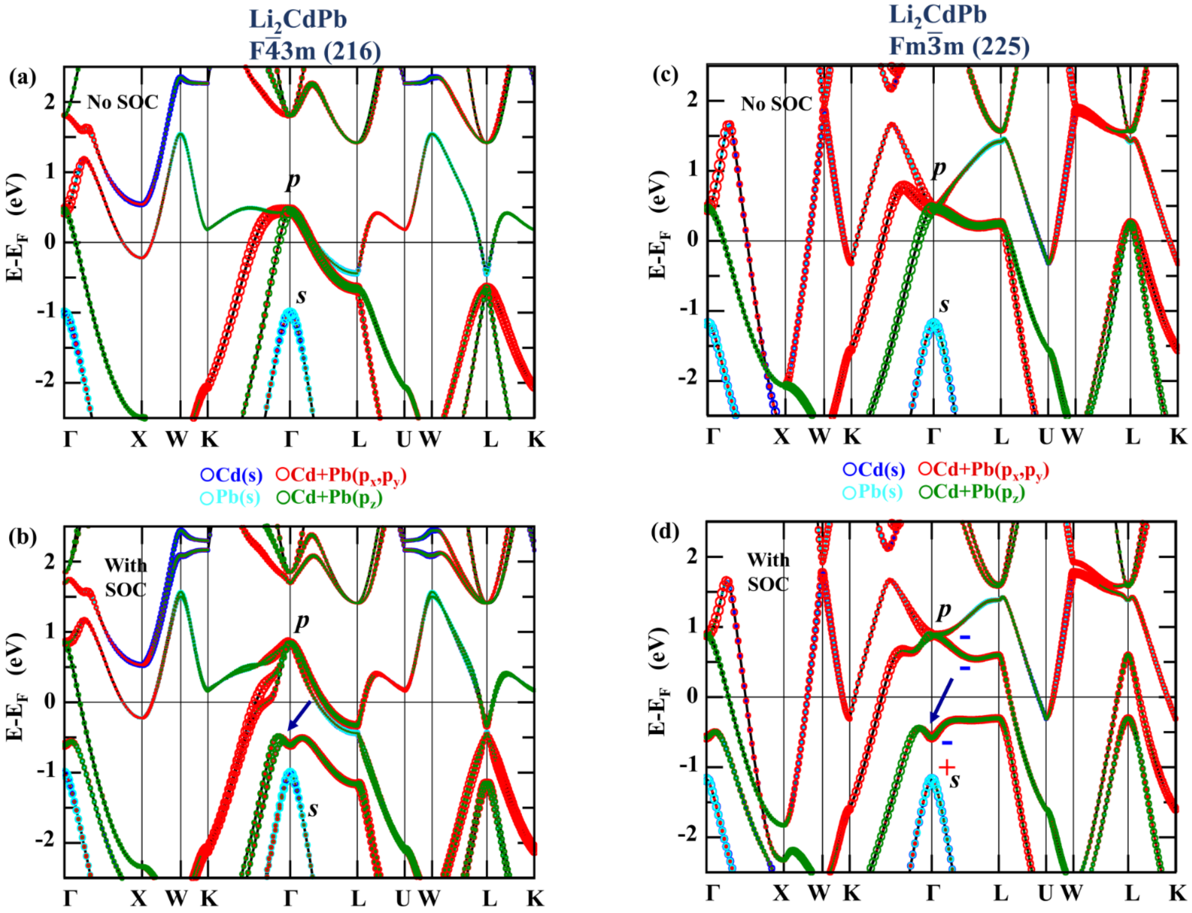

Figure S12. The orbital band projections of Li_2CdPb (a) without SOC and (b) with SOC under space group $F\bar{4}3m$ (216). The orbital band projections of Li_2CdPb (c) without SOC and (d) with SOC under space group $Fm\bar{3}m$ (225). (d) Blue sign (-) represents odd parity and red sign (+) shows even parity. The blue arrow indicates an inversion in the band under SOC.

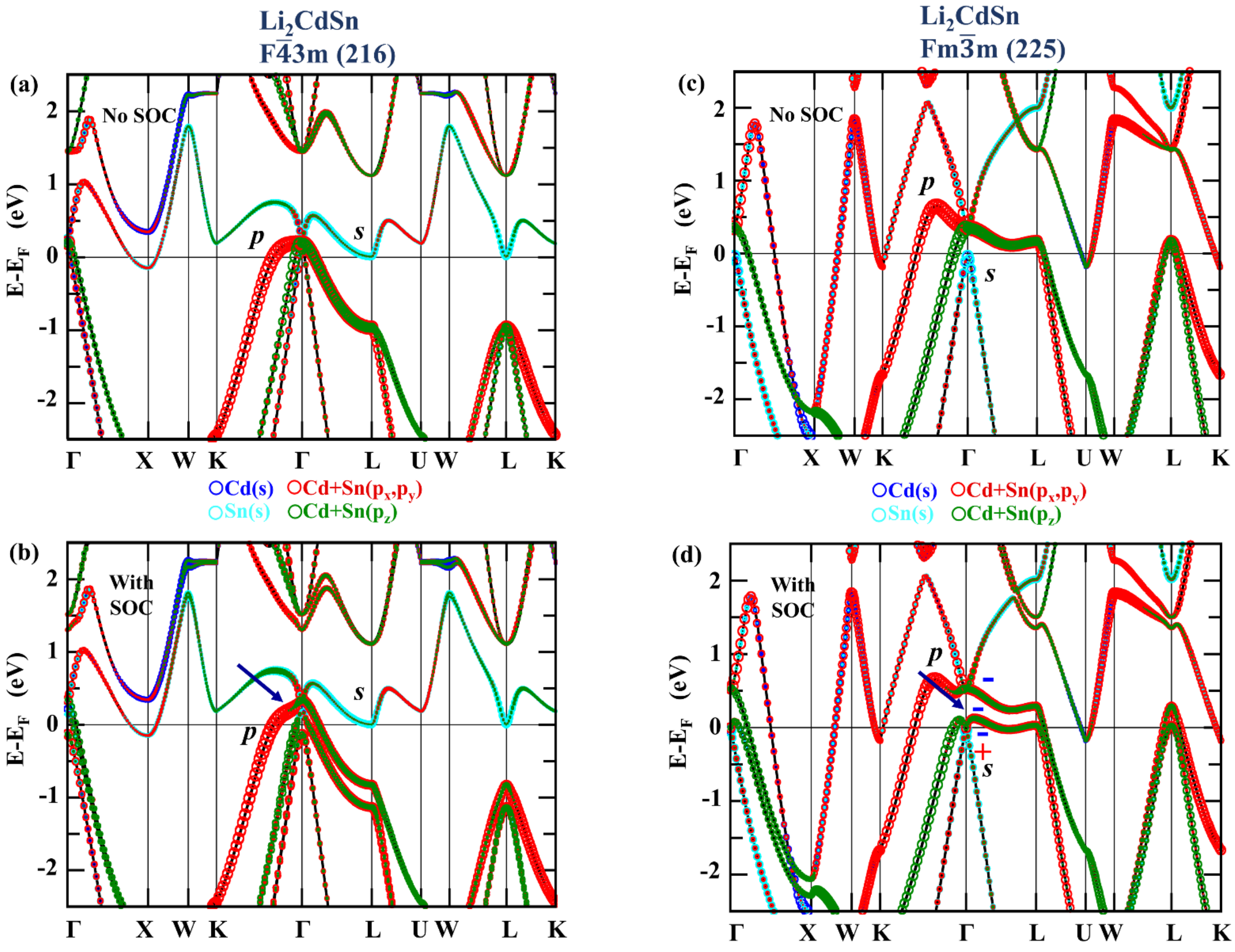

Figure S13. The orbital band projections of Li_2CdSn (a) without SOC and (b) with SOC under space group F$\bar{4}$3m (216). The orbital band projections of Li_2CdSn (c) without SOC and (d) with SOC under space group Fm$\bar{3}$m (225). (d) Blue sign (-) represents odd parity and red sign (+) shows even parity. The blue arrow indicates an inversion in the band under SOC.

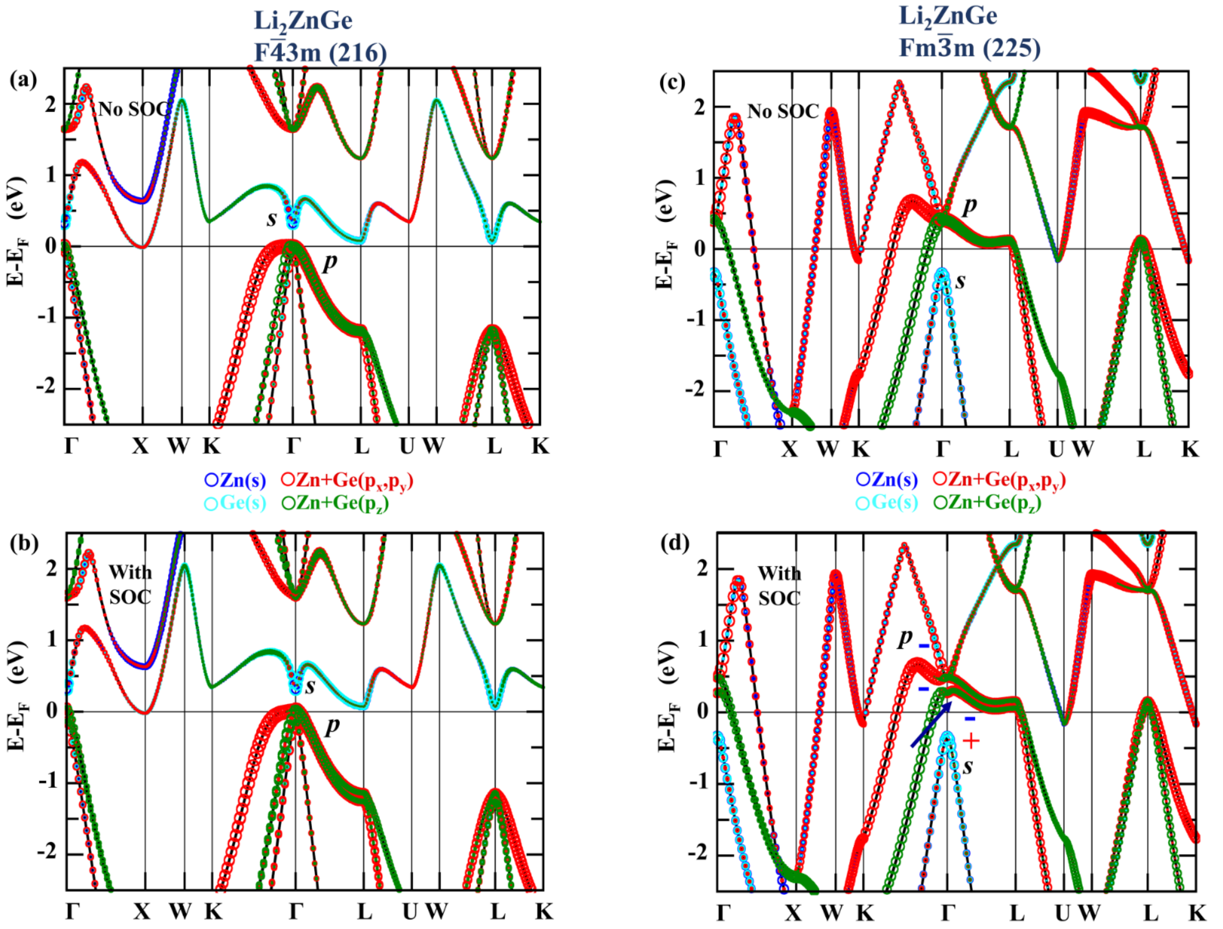

Figure S14. The orbital band projections of Li_2ZnGe (a) without SOC and (b) with SOC under space group F$\bar{4}$3m (216). The orbital band projections of Li_2ZnGe (c) without SOC and (d) with SOC under space group Fm$\bar{3}$m (225). (d) Blue sign (-) represents odd parity and red sign (+) shows even parity. The blue arrow indicates an inversion in the band under SOC.

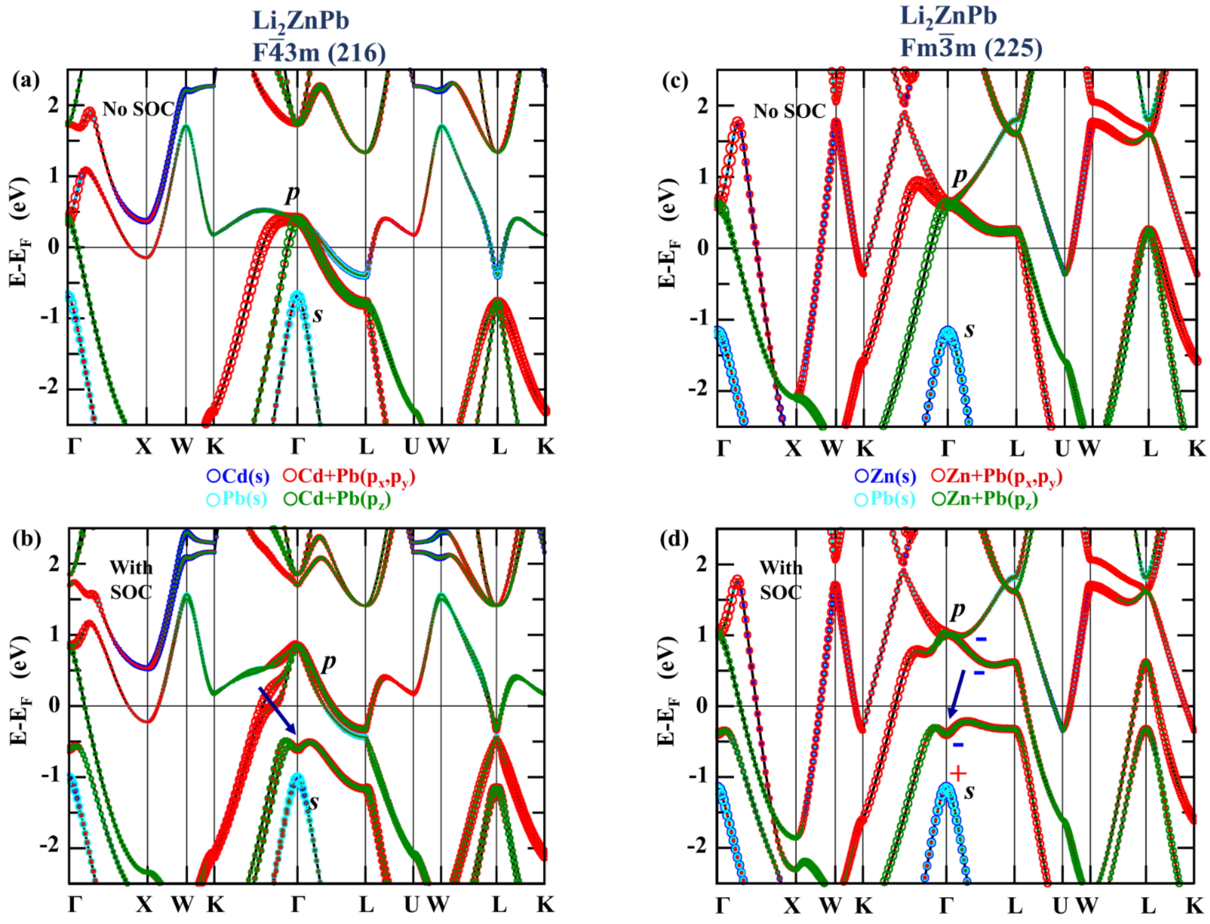

Figure S15. The orbital band projections of Li_2ZnPb (a) without SOC and (b) with SOC under space group F$\bar{4}$3m (216). The orbital band projections of Li_2ZnPb (c) without SOC and (d) with SOC under space group Fm$\bar{3}$m (225). (d) Blue sign (-) represents odd parity and red sign (+) shows even parity. The blue arrow indicates an inversion in the band under SOC.

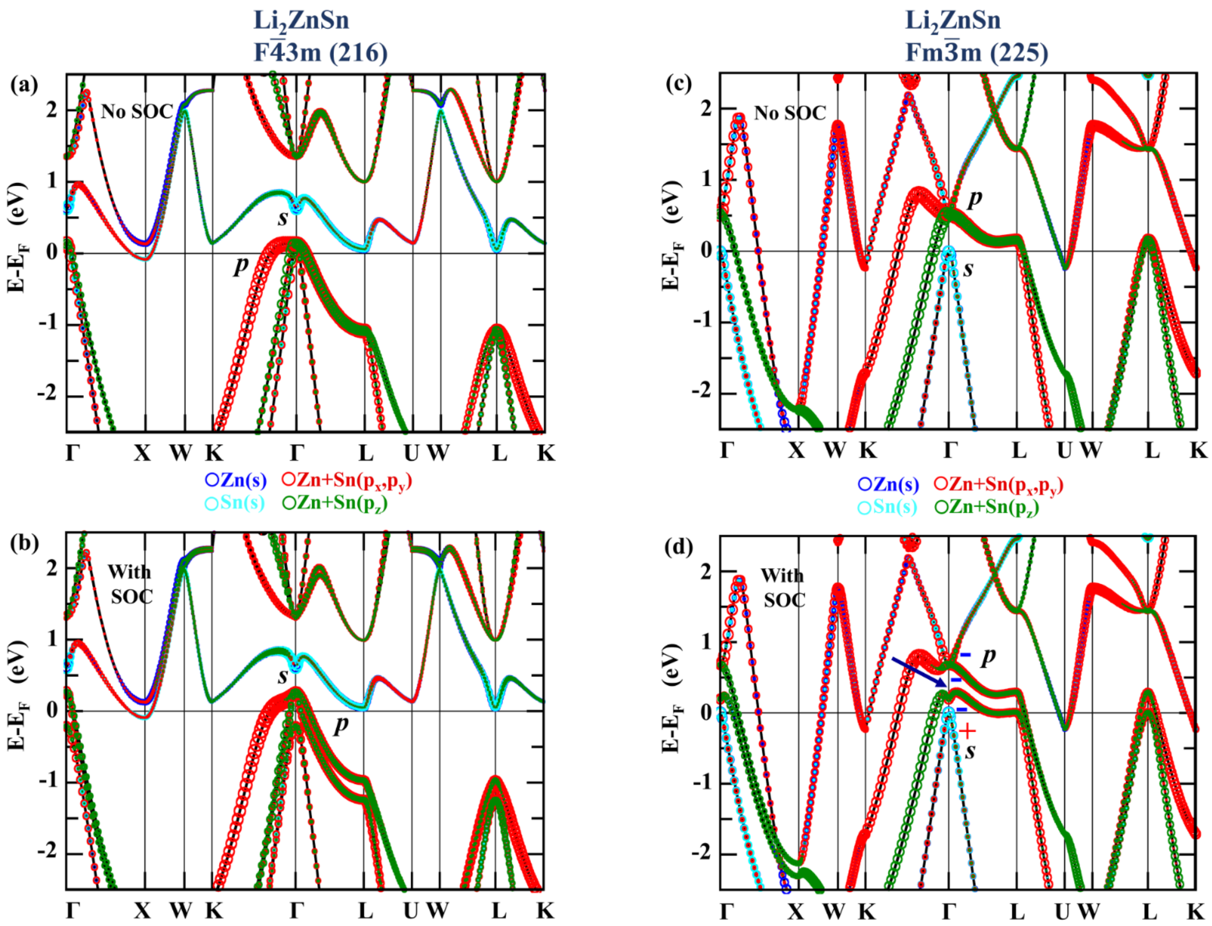

Figure S16. The orbital band projections of Li_2ZnSn (a) without SOC and (b) with SOC under space group F$\bar{4}$3m (216). The orbital band projections of Li_2ZnSn (c) without SOC and (d) with SOC under space group Fm$\bar{3}$m (225). (d) Blue sign (-) represents odd parity and red sign (+) shows even parity. The blue arrow indicates an inversion in the band under SOC.

2.3. Symmetry analysis and surface states of Li_2CdGe in space group $Fm\overline{3}m$

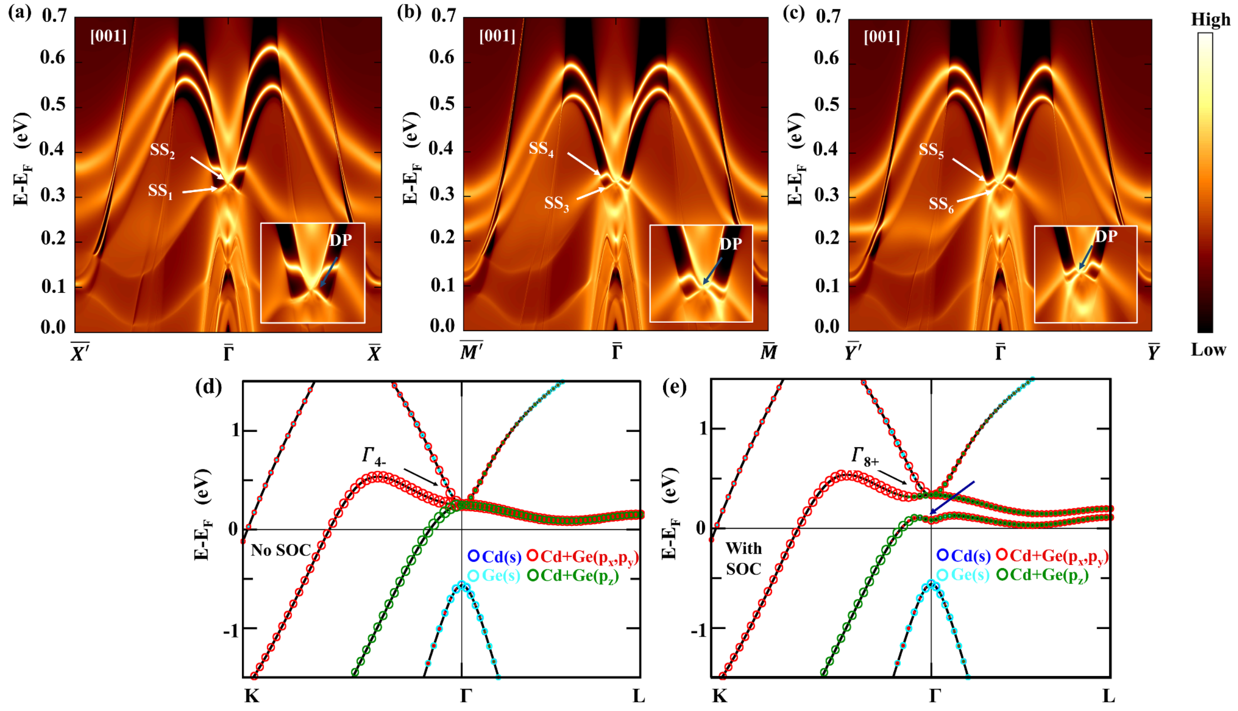

Figure S17. Surface electronic spectrum of Li_2CdGe showing the topologically protected surface states along (a) $\overline{X}'$-$\overline{\Gamma}$- $\overline{X}$, (b) $\overline{M}'$-$\overline{\Gamma}$- $\overline{M}$ path, and (c) $\overline{Y}'$-$\overline{\Gamma}$- $\overline{Y}$ paths on the 001 plane of the BZ. SS shows surface states. DP represents the Dirac point. The zoomed-in inset represents a clear intersection of SS from valence and conduction bands, generating the Dirac point. Orbital projected band structure of Li_2CdGe in $Fm\overline{3}m$ (225) phase for (d) without SOC and (e) with SOC, with irreducible representation, under PBE-GGA. Blue and cyan circles represent *s* orbitals from Cd and Ge, respectively; red circles represent p_x and p_y states; green circles represent p_z states from Cd and Ge, respectively. The black arrows represent irreducible representations at Γ stabilizing TP/DP. The blue arrow shows band inversion after SOC.

2.4. Surface states of Li_2CdPb in space group Fm$\bar{3}$m

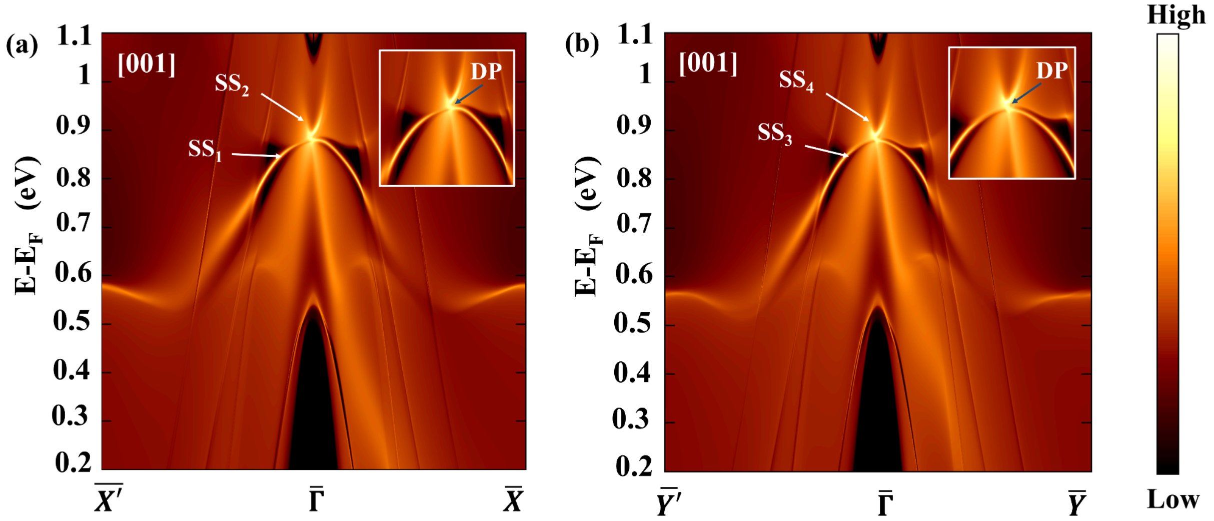

Figure S18. Surface electronic spectra of Li_2CdPb in Fm$\bar{3}$m phase along (a) $\overline{X}$'-$\overline{\Gamma}$- $\overline{X}$ and (b) $\overline{Y}$'-$\overline{\Gamma}$- $\overline{Y}$ paths on the 001 plane of the Brillouin zone. SS shows surface states. DP represents the Dirac point. The zoomed-in inset represents a clear intersection of SS from valence and conduction bands, giving the Dirac point.

3. Evidence of Topological Phase by Parity Analysis, Wannier Charge Centers Evolution and SymTopo Analysis

3.1. Parity analysis of Li_2YZ in $Fm\bar{3}m$ phases.

Table S3. The parities of all the occupied bands of Li_2CdGe in space group $Fm\bar{3}m$ (225) at all the TRIM points in the Brillouin zone.

**Band Index**	**X**	**X**	**X**	**Γ**	**L**	**L**	**L**	**L**	**Total**
34	-	-	-	-	-	-	-	-	+
32	-	-	-	-	+	+	+	+	+
30	+	+	+	-	+	+	+	+	-
28	-	-	-	+	+	+	+	+	-
26	+	+	+	+	-	-	-	-	+
24	+	+	+	+	+	+	+	+	+
22	+	+	+	+	+	+	+	+	+
20	+	+	+	+	+	+	+	+	+
18	+	+	+	+	+	+	+	+	+
16	+	+	+	+	+	+	+	+	+
14	+	+	+	+	-	-	-	-	+
12	+	+	+	+	-	-	-	-	+
10	+	+	+	+	-	-	-	-	+
8	+	+	+	+	-	-	-	-	+
6	+	+	+	+	-	-	-	-	+
4	+	+	+	-	-	-	-	-	-
2	+	+	+	+	+	+	+	+	+
**Total**	**-**	**-**	**-**	+	+	+	+	+	**(-)**

Table S4. The parities of all the occupied bands of Li_2CdPb in space group $Fm\bar{3}m$ (225) at all the TRIM points in the Brillouin zone.

**Band Index**	**X**	**X**	**X**	**Γ**	**L**	**L**	**L**	**L**	**Total**
34	-	-	-	-	-	-	-	-	+
32	-	-	-	-	+	+	+	+	+
30	+	+	+	-	+	+	+	+	-
28	-	-	-	+	+	+	+	+	-
26	+	+	+	+	+	+	+	+	+
24	+	+	+	+	+	+	+	+	+
22	+	+	+	+	+	+	+	+	+
20	+	+	+	+	-	-	-	-	+
18	+	+	+	+	+	+	+	+	+
16	+	+	+	+	+	+	+	+	+
14	+	+	+	+	-	-	-	-	+
12	+	+	+	+	-	-	-	-	+
10	+	+	+	+	-	-	-	-	+
8	+	+	+	+	-	-	-	-	+
6	+	+	+	+	-	-	-	-	+
4	+	+	+	-	-	-	-	-	-
2	+	+	+	+	+	+	+	+	+
**Total**	-	-	-	+	+	+	+	+	**(-)**

Table S5. The parities of all the occupied bands of Li_2CdSn in space group Fm$\bar{3}$m (225) at all the TRIM points in the Brillouin zone.

**Band Index**	**X**	**X**	**X**	**Γ**	**L**	**L**	**L**	**L**	**Total**
34	-	-	-	-	-	-	-	-	+
32	-	-	-	-	+	+	+	+	+
30	+	+	+	-	+	+	+	+	-
28	-	-	-	+	+	+	+	+	-
26	+	+	+	+	-	-	-	-	+
24	+	+	+	+	+	+	+	+	+
22	+	+	+	+	+	+	+	+	+
20	+	+	+	+	+	+	+	+	+
18	+	+	+	+	+	+	+	+	+
16	+	+	+	+	+	+	+	+	+
14	+	+	+	+	-	-	-	-	+
12	+	+	+	+	-	-	-	-	+
10	+	+	+	+	-	-	-	-	+
8	+	+	+	+	-	-	-	-	+
6	+	+	+	+	-	-	-	-	+
4	+	+	+	-	-	-	-	-	-
2	+	+	+	+	+	+	+	+	+
**Total**	-	-	-	+	+	+	+	+	**(-)**

Table S6. The parities of all the occupied bands of Li_2ZnGe in space group $Fm\bar{3}m$ (225) at all the TRIM points in the Brillouin zone.

**Band Index**	**X**	**X**	**X**	**Γ**	**L**	**L**	**L**	**L**	**Total**
34	-	-	-	-	-	-	-	-	+
32	-	-	-	-	+	+	+	+	+
30	+	+	+	-	+	+	+	+	-
28	-	-	-	+	+	+	+	+	-
26	+	+	+	+	+	+	+	+	+
24	+	+	+	+	+	+	+	+	+
22	+	+	+	+	+	+	+	+	+
20	+	+	+	+	+	+	+	+	+
18	+	+	+	+	+	+	+	+	+
16	+	+	+	+	-	-	-	-	+
14	+	+	+	+	-	-	-	-	+
12	+	+	+	+	-	-	-	-	+
10	+	+	+	+	-	-	-	-	+
8	+	+	+	+	-	-	-	-	+
6	+	+	+	+	-	-	-	-	+
4	+	+	+	-	-	-	-	-	-
2	+	+	+	+	+	+	+	+	+
**Total**	-	-	-	+	+	+	+	+	**(-)**

Table S7. The parities of all the occupied bands of Li_2ZnPb in space group Fm$\overline{3}$m (225) at all the TRIM points in the Brillouin zone.

**Band Index**	**X**	**X**	**X**	**Γ**	**L**	**L**	**L**	**L**	**Total**
34	-	-	-	-	-	-	-	-	+
32	-	-	-	-	+	+	+	+	+
30	+	+	+	-	+	+	+	+	-
28	-	-	-	+	+	+	+	+	-
26	+	+	+	+	+	+	+	+	+
24	+	+	+	+	+	+	+	+	+
22	+	+	+	+	+	+	+	+	+
20	+	+	+	+	+	+	+	+	+
18	+	+	+	+	+	+	+	+	+
16	+	+	+	+	-	-	-	-	+
14	+	+	+	+	-	-	-	-	+
12	+	+	+	+	-	-	-	-	+
10	+	+	+	+	-	-	-	-	+
8	+	+	+	+	-	-	-	-	+
6	+	+	+	+	-	-	-	-	+
4	+	+	+	-	-	-	-	-	-
2	+	+	+	+	+	+	+	+	+
**Total**	-	-	-	+	+	+	+	+	**(-)**

Table S8. The parities of all the occupied bands of Li_2ZnSn in space group $Fm\bar{3}m$ (225) at all the TRIM points in the Brillouin zone.

**Band Index**	**X**	**X**	**X**	**Γ**	**L**	**L**	**L**	**L**	**Total**
34	-	-	-	-	-	-	-	-	+
32	-	-	-	-	+	+	+	+	+
30	+	+	+	-	+	+	+	+	-
28	-	-	-	+	+	+	+	+	-
26	+	+	+	+	+	+	+	+	+
24	+	+	+	+	+	+	+	+	+
22	+	+	+	+	+	+	+	+	+
20	+	+	+	+	+	+	+	+	+
18	+	+	+	+	+	+	+	+	+
16	+	+	+	+	-	-	-	-	+
14	+	+	+	+	-	-	-	-	+
12	+	+	+	+	-	-	-	-	+
10	+	+	+	+	-	-	-	-	+
8	+	+	+	+	-	-	-	-	+
6	+	+	+	+	-	-	-	-	+
4	+	+	+	+	-	-	-	-	+
2	+	+	+	-	+	+	+	+	-
**Total**	-	-	-	+	+	+	+	+	**(-)**

3.2. Wannier charge center plots of Li_2CdPb in $Fm\bar{3}m$ phase.

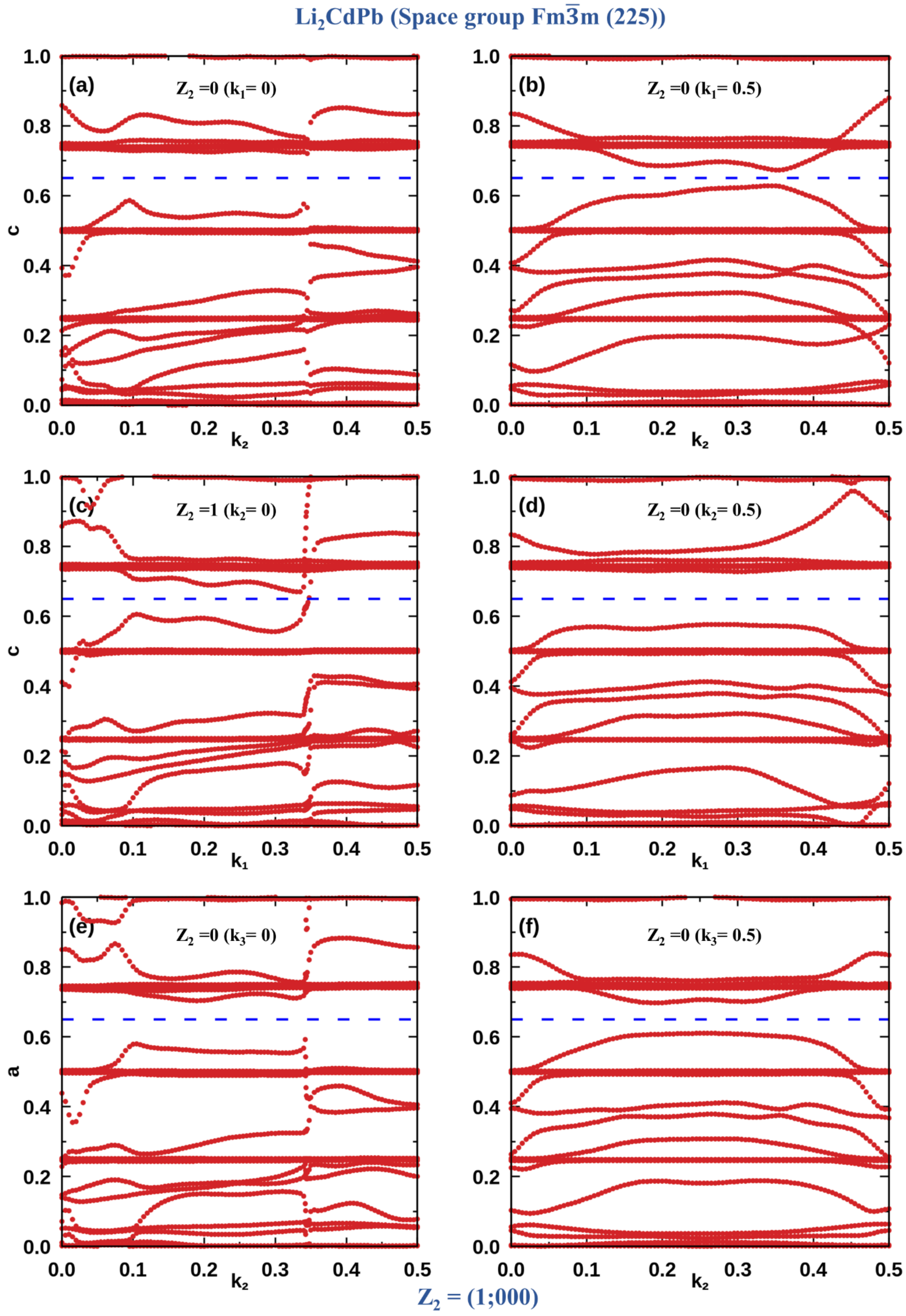

Figure S19. Evolution of Wannier charge centers (WCCs) for Li_2CdPb in space group $Fm\bar{3}m$ (225), calculated for the six time-reversal-invariant planes in the Brillouin zone: (a) $k_1 = 0$, (b) $k_1 = 0.5$, (c) $k_2 = 0$, (d) $k_2 = 0.5$, (e) $k_3 = 0$, and (f) $k_3 = 0.5$. The calculated Z_2 invariants for each plane are indicated in the corresponding panels. The blue line represents the reference line.

3.3. SymTopo Analysis

Table S9. Detailed investigation of the topological properties of Li_2YZ compounds in two structural phases under PBE-GGA, without and with the inclusion of SOC. HSPSM denotes a high-symmetry-point semimetal, SM indicates a normal semimetal, and NI represents a normal insulator.

Materials	Space group	SOC	Topological Classification	Kpoint Degenerate Bands (Degeneracy)	Irreducible Representation (Irreps)
Li_2CdGe	$F\bar{4}3m$	No	HSPSM	Γ 15-17 (3)	Γ_{4+}
		Yes	HSPSM	Γ 31-34 (4)	Γ_{8+}
	$Fm\bar{3}m$	No	HSPSM	Γ 15-17 (3); X 16-17 (2)	Γ_{4-} ; X_{5-}
		Yes	HSPSM	Γ 31-34 (4)	Γ_{8+}
Li_2CdPb	$F\bar{4}3m$	No	HSPSM	Γ 15-17 (3)	Γ_{4+}
		Yes	HSPSM	Γ 31-34 (4)	Γ_{8+}
	$Fm\bar{3}m$	No	HSPSM	Γ 15-17 (3) ; X 16-17 (2)	Γ_{4-} ; X_{5-}
		Yes	HSPSM	Γ 31-34 (4)	Γ_{8+}
Li_2CdSn	$F\bar{4}3m$	No	SM	--	--
		Yes	HSPSM	Γ 31-34 (4)	Γ_{8+}
	$Fm\bar{3}m$	No	HSPSM	Γ15-17 (3) ; X 16-17 (2)	Γ_{4-} ; X_{5-}
		Yes	HSPSM	Γ 31-34 (4)	Γ_{8+}
Li_2ZnGe	$F\bar{4}3m$	No	NI	--	--
		Yes	NI	--	--
	$Fm\bar{3}m$	No	HSPSM	Γ 15-17 (3) ; X 16-17 (2)	Γ_{4-} ; X_{5-}
		Yes	HSPSM	Γ 31-34 (4)	Γ_{8+}
Li_2ZnPb	$F\bar{4}3m$	No	HSPSM	Γ 15-17 (3)	Γ_{4+}
		Yes	HSPSM	Γ 31-34 (4)	Γ_{8+}
	$Fm\bar{3}m$	No	HSPSM	Γ 15-17 (3) ; X 16-17 (2)	Γ_{4-} ; X_{5-}
		Yes	HSPSM	Γ 31-34 (4)	Γ_{8+}
Li_2ZnSn	$F\bar{4}3m$	No	NI	--	--
		Yes	NI	--	--
	$Fm\bar{3}m$	No	HSPSM	Γ 15-17 (3) ; X 16-17 (2)	Γ_{4-} ; X_{5-}
		Yes	HSPSM	Γ 31-34 (4)	Γ_{8+}

4. KPOINTS and Coordinates of Bulk Brillouin Zone

4.1. KPOINTS

```
KPOINTS
  0
Gamma
9    9    9
0.0  0.0  0.0
```

4.2. Coordinates of the bulk Brillouin zone

```
System = Cubic K-path
10
Line mode
Reciprocal
  0.0000000000    0.0000000000    0.0000000000   !Gamma
  0.5000000000    0.0000000000    0.5000000000   !X

  0.5000000000    0.0000000000    0.5000000000   !X
  0.5000000000    0.2500000000    0.7500000000   !W

  0.5000000000    0.2500000000    0.7500000000   !W
  0.3750000000    0.3750000000    0.7500000000   !K

  0.3750000000    0.3750000000    0.7500000000   !K
  0.0000000000    0.0000000000    0.0000000000   !Gamma

  0.0000000000    0.0000000000    0.0000000000   !Gamma
```

0.5000000000	0.5000000000	0.5000000000	!L
0.5000000000	0.5000000000	0.5000000000	!L
0.6250000000	0.2500000000	0.6250000000	!U
0.6250000000	0.2500000000	0.6250000000	!U
0.5000000000	0.2500000000	0.7500000000	!W
0.5000000000	0.2500000000	0.7500000000	!W
0.5000000000	0.5000000000	0.5000000000	!L
0.5000000000	0.5000000000	0.5000000000	!L
0.3750000000	0.3750000000	0.7500000000	!K
